# Neurotrophic factor-α1/carboxypeptidase E controls progression and reversal of Alzheimer's disease pathogenesis in mice

**DOI:** 10.7150/thno.99908

**Published:** 2025-01-13

**Authors:** Fang-Cheng Fan, Li-Ming Liu, Mei Guo, Yang Du, Yue-Wen Chen, Y. Peng Loh, Yong Cheng

**Affiliations:** 1 Department of Psychiatry, The Third People's Hospital of Foshan, Foshan, Guangdong, China, 528000; 2 Key Laboratory of Ethnomedicine of Ministry of Education, Center for Translational Neuroscience, School of Pharmacy, Minzu University of China, 27 Zhongguancun South St, Haidian District, Beijing, China, 100081.; 3 Institute of National Security, Minzu University of China, 27 Zhongguancun South St, Haidian District, Beijing, China, 100081.; 4 Chinese Academy of Sciences Key Laboratory of Brain Connectome and Manipulation, Shenzhen Key Laboratory of Translational Research for Brain Diseases, The Brain Cognition and Brain Disease Institute, Shenzhen Institute of Advanced Technology, Chinese Academy of Sciences; Shenzhen-Hong Kong Institute of Brain Science—Shenzhen Fundamental Research Institutions, 1068 Xueyuan Avenue, Xili Shenzhen University City, Nanshan District, Shenzhen, Guangdong, China, 518055.; 5 Guangdong Provincial Key Laboratory of Brain Science, Disease and Drug Development, HKUST Shenzhen Research Institute, 9 Yuexing 1st Road, Nanshan District, Shenzhen, Guangdong, China, 518057.; 6 Section on Cellular Neurobiology, Eunice Kennedy Shriver National Institute of Child Health and Human Development, National Institutes of Health, 9000 Rockville Pike, Bethesda, Maryland, United States of America, 20892.

**Keywords:** Neurotrophic Factor-α1/Carboxypeptidase E (NF-α1/CPE), Alzheimer's Disease (AD), 5xFAD Mouse, Neuroprotection, Therapeutic Target

## Abstract

**Background:** Neurotrophic Factor-α1/Carboxypeptidase E (NF-α1/CPE) is a pivotal neuroprotective protein implicated in rescuing cognitive decline associated with Alzheimer's disease (AD). However, its direct role in AD pathogenesis remains unexplored.

**Methods:** We utilized the Cre/LoxP system to diminish NF-α1/CPE expression, and employed AAV-mediated overexpression of NF-α1/CPE.

**Results:** NF-α1/CPE expression was significantly down-regulated in advanced stages of AD and with age in 5xFAD mice. Reduced NF-α1/CPE levels in the hippocampus of 5xFAD mice increased plaque burden, microglial cell count, disrupted synaptogenesis, and intensified cognitive impairments at 5 and 7 months. However, by 9 months, no further progression of detrimental effects was observed. Overexpression of NF-α1/CPE markedly decreased amyloid plaque accumulation, mitigated spatial memory deficits, and normalized hippocampal synaptogenesis and microglial anomalies across early and late stages of the disease.

**Conclusion:** NF-α1/CPE is a critical regulator of AD pathogenesis, offering promising therapeutic potential for reducing amyloid beta deposition and toxicity in AD.

## Introduction

Alzheimer's Disease (AD) is the most prevalent neurodegenerative disease among the aging population globally 1. AD is marked by cognitive dysfunction, notably memory loss, as well as extracellular deposition of aggregated amyloid and intracellular tau neurofibrillary tangles 2. Environmental factors, stress and aging have been proposed to contribute to the development of AD 3,4. A small number of cases (1-5%) are attributed to genetic inheritance of one of three mutant genes: (*APP*), presenilin 1 (*PS1*), and presenilin 2 (*PS2*) 5. At the present time, there is no cure for the disease 6. Therapeutic targets for AD drug development have focused on the clearance of amyloid plaques which includes ones that up-regulate expression of degradative enzymes 7 and cholinesterase inhibitors 8. Other strategies are directed towards targeting tau and involve the use of anti-tau antibodies 9,10 and an anti-tau vaccine 11. These pharmacological agents have provided some improvement of cognitive function in patients but did not slow progression of the disease 12. Delivery of neurotrophic factor genes such as nerve growth factor (NGF) to the brain have also shown some improvement of cognitive decline 13,14. In pre-clinical studies, hippocampal injection of lenti- or Adeno-associated virus (AAV) carrying brain derived neurotrophic factor (BDNF) 15 or fibroblast growth factor (FGF2) 15,16, respectively, have ameliorated cognitive deficits in AD mouse models, independent of direct modulation of amyloid expression, but by partially increasing clearance of amyloid β peptide and enhancing neurogenesis 17. A clinical trial involving BDNF gene therapy is now in progress to determine its efficacy in treating AD patients 18. Another neurotrophic factor, Neurotrophic Factor-α1/Carboxypeptidase E (NF-α1/CPE) which has strong neuroprotective activity 19-21 has been shown to prevent the subsequent development of AD pathology and memory loss in pre-symptomatic 3xTg-AD mice 22. Unlike the other neurotrophic factors studied, NF-α1/CPE down-regulated expression of amyloid precursor protein (APP) at the transcriptional and translational level and attenuated insoluble Aβ1-42 production. Moreover, AAV-NF-α1/CPE treatment of 3xTg-AD mice increased expression of Bcl2, and decreased expression of Bax. Tau hyper-phosphorylation and neurodegeneration were prevented, and reactive microglia numbers were reduced, as was pro-inflammatory protein Card14, in NF-α1/CPE treated versus untreated 3xTg mice. Administration of a combination of neurotrophic factors may be potentially a good strategy for treating AD.

Studies have shown that NF-α1/CPE levels decline with age in the brain of mice 23. It has also been reported that diminished levels of NF-α1/CPE in the subventricular zone (SVZ) in old mice resulted in decreased neurogenesis 23,24. Interestingly, intracerebral infusion of NF-α1/CPE or injection of lentivirus expressing CPE into the lateral ventricle of middle aged or old mice resulted in enhancement of neurogenesis 23. Conversely, injection of CPE-shRNA into the SVZ to down-regulate NF-α1/CPE expression impaired neurogenesis, indicating that it is a positive regulator of adult neurogenesis and may mitigate AD progression 23. In the hippocampus, NF-α1/CPE is highly expressed in the CA3 region and plays a critical role in maintaining the survival of adult CA3 neurons and normal cognitive function. Specifically, CPE knockout (KO) mice exhibit deficits in memory consolidation, synaptic physiology, and CA3 pyramidal neuron degeneration 25. Additionally, CPE has been shown to exert neuroprotective and antidepressant activities, as well as regulate stem cell differentiation in the central nervous system 20.

A report indicated that overexpression of NF-α1/CPE can prevent development AD pathogenesis 22 suggest that there might be a direct causal relationship between its decreased levels in neurons and the onset and progression of AD. In this study, we examined whether the decline of NF-α1/CPE levels with age and genetic knock down of expression in a 5xFAD mouse model, is directly correlated with onset, progression and severity of AD pathogenesis. 5xFAD mice exhibit amyloid pathology with plaques in the brain from 2 months of age 26,27. Microgliosis and neuroinflammation, as well as synaptic and neuronal losses are observed by 5 months of age 26-29. To determine if NF-α1/CPE controls AD pathogenesis, we specifically diminished neuronal expression of NF-α1/CPE utilizing a Cre/LoxP in the hippocampus of 5xFAD mice at various ages (5, 7 and 9 months). We then examined the increase in amyloid plaques and other AD -related pathology. Finally, we increased the level of NF-α1/CPE in 5xFAD mice at ages 5, 7 and 9 months by injection of AAV-NF-α1/CPE in the hippocampus and analyzed for reversal of AD pathogenesis.

## Materials and Methods

### Animals

C57BL/6 mice were obtained from the Vital River Laboratory, Beijing, China. The 5×FAD mice (provided by Academy of Military Medical Sciences), Camk2a-Cre mice were crossed with CPE^flox/flox^ mice (purchased from Cyagen Biosciences, Suzhou, China), respectively. All animals were maintained under specific pathogen-free (SPF) conditions, at ambient temperatures (24 °C ± 2 °C), following a 12 h light/12 h dark cycle with unrestricted access to standard rodent chow and clean water. Mouse genotyping protocols are in *SI Appendix Materials and Methods*. All experimental procedures involving the animals were sanctioned by the Experimental Animal Ethics Committee at Minzu University of China.

### Stereotactic injection

AAV-CPE and a control AAV-Empty were generated by co-transfection of an AAV shuttle vector (obtained from Syngenbio, Beijing, China). Following administration of 10% pentobarbital for anesthesia and immobilization on a stereotaxic frame, each animal received a 0.5 μl injection of AAV (1 × 10^10^ vg/ mL) into both hippocampi using the coordinates AP, -2.1; ML, ±1.7; and DV, -2.1. The syringe was then kept in place for an additional 10 min to prevent any reverse flow. The mice were bilaterally injected with either AAV-Empty or AAV-CPE at the age of 2 months and subjected to behavioral tests at 5, 7, and 9 months of age. For Pearson correlation analysis, mice received unilateral injections of AAV-Empty or AAV-CPE at 2 months of age and harvested 3 months later.

### Behavioral tests

#### Morris Water Maze test

The Morris Water Maze (MWM) test was employed to evaluate the cognitive abilities of mice, as previously described 22. Details provided in *SI Appendix Materials and Methods*.

#### Y maze

The Y-maze test was used to assess short term memory following a previously published protocol 19. Details provided in *SI Appendix Materials and Methods*.

#### Novel object recognition test

The Novel object recognition test was used to evaluate recognition memory following our published protocol 19. Details provided in *SI Appendix Materials and Methods*.

### Quantitative RT-PCR

Total RNA was isolated from brain tissues using TRIzol reagent. One microgram of RNA was utilized in a one-step first-strand cDNA synthesis kit (Genstar Biotech, Beijing, China). Quantitative real-time (RT) polymerase chain reaction (PCR) was conducted using 2× SYBR Green PCR master mix (Genstar Biotech). The primers utilized for analysis are listed in Table S1 in *SI Appendix Materials and Methods*.

### Immunofluorescence staining

All procedures were conducted as our previously described study 19. The antibodies used are provided in *SI Appendix Materials and Methods.*

### Golgi-Cox staining

Golgi-Cox staining of hippocampal tissues was carried out using the FD Rapid Golgi Stain™ Kit (FD NeuroTechnologies, Shanghai, China) according to the manufacturer's protocol. Details provided in *SI Appendix Materials and Methods.*

### Western blotting

Western blotting was carried out according to previously published protocol 19. Details of the procedure and antibodies used are in *SI Appendix Materials and Methods.*

### Statistical analysis

Data were subjected to student's t-tests for comparisons between two groups, while one-way or two-way analysis of variance (ANOVA) was employed for comparisons involving multiple groups. The analysis was conducted using GraphPad Prism 8.0 (Prism GraphPad software, La Jolla, USA). All values are presented as means ± standard deviation (SD). A significance level of p < 0.05 was deemed as statistically significant.

## Results

### NF-α1/CPE diminishes with age in hippocampus of wild-type and 5xFAD mice

**Figure 1:**
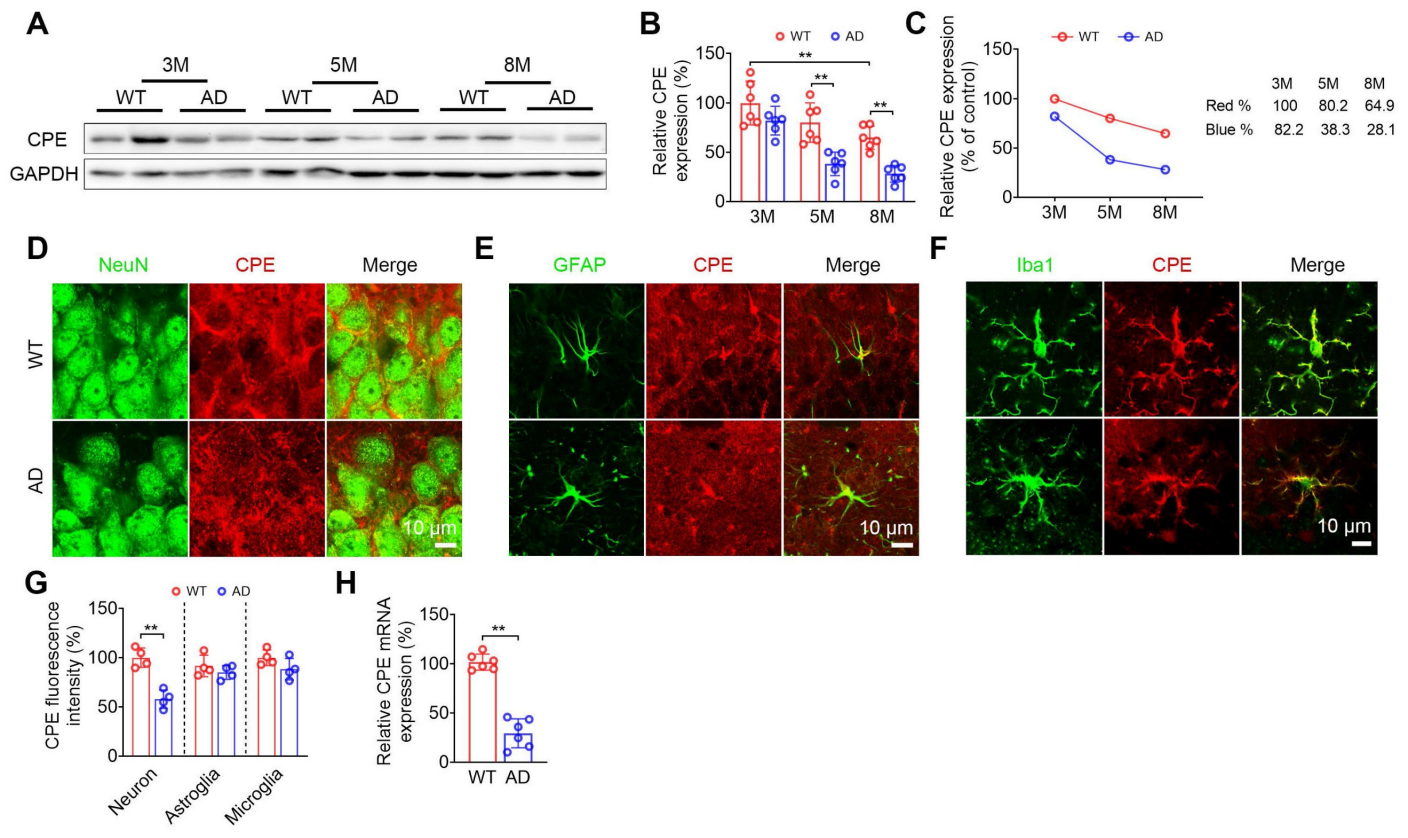
The levels of CPE are reduced in AD mice. (A) Western blot analysis indicates the levels of CPE in the hippocampus of wild type (WT) and 5×FAD mice at 3 months, 5 months, and 8 months old. (B) CPE levels were normalized to GAPDH. n = 6 mice, One-way ANOVA analysis followed by Tukey's post-hoc multiple comparison test, [F (5,30) = 18.80, p < 0.0001; 3M: WT vs. 8M: WT, p = 0.0061; 5M: WT vs. 5M: AD, p = 0.0008; 8M: WT vs. 8M: AD, p = 0.0039]. (C) Time course of hippocampal depletion of CPE with age in WT and 5xFAD mice. (D-G) Coexpression (NeuN and CPE; GFAP and CPE; Iba1 and CPE;) and quantification of CPE in the hippocampal CA3 region of 5-month-old wild type and 5×FAD mice. n = 4 mice, average of 4-6 slices per mouse, average of n=50 to 70 for neuron+ positive cells, n=20 to 30 for GFAP+ and Iba1+ positive cells per slice, Unpaired t test, [Neuron: p = 0.0008; Astroglia: p = 0.3432; Microglia: p = 0.1359]. (H) RT-PCR was conducted to examine CPE mRNA expression in the hippocampus of 5-month-old 5×FAD mice. Data were standardized to the control group. n = 6 mice, Unpaired t test, [p < 0.0001].

The change in hippocampal NF-α1/CPE level was determined in wild-type (WT) and 5xFAD mice at 3, 5 and 8 mths of age (Figure 1). WT mice showed a decrease of NF-α1/CPE protein with age and was significant by 8 mths of age (Figure 1A-C). In contrast, the loss of NF-α1/CPE was much more severe in 5xFAD mice during those ages, with a significant decrease to ~33% of control (3 mth WT) by 5 mths of age (Figure 1C). Loss of NF-α1/CPE protein was significant in neurons, but not in microglia and astroglia at 5mths (Figure 1D-G). The greater loss of NF-α1/CPE in 5xFAD mice compared to WT was also reflected at the mRNA level at 5 mths (Figure 1H). Moreover, the loss of NF-α1/CPE was correlated with the loss of neurons in AD mice (Figure S1).

### Neuronal NF-α1/CPE knock-down impairs cognitive functions, increases Aβ deposition and microgliosis in 5xFAD mice

**Figure 2:**
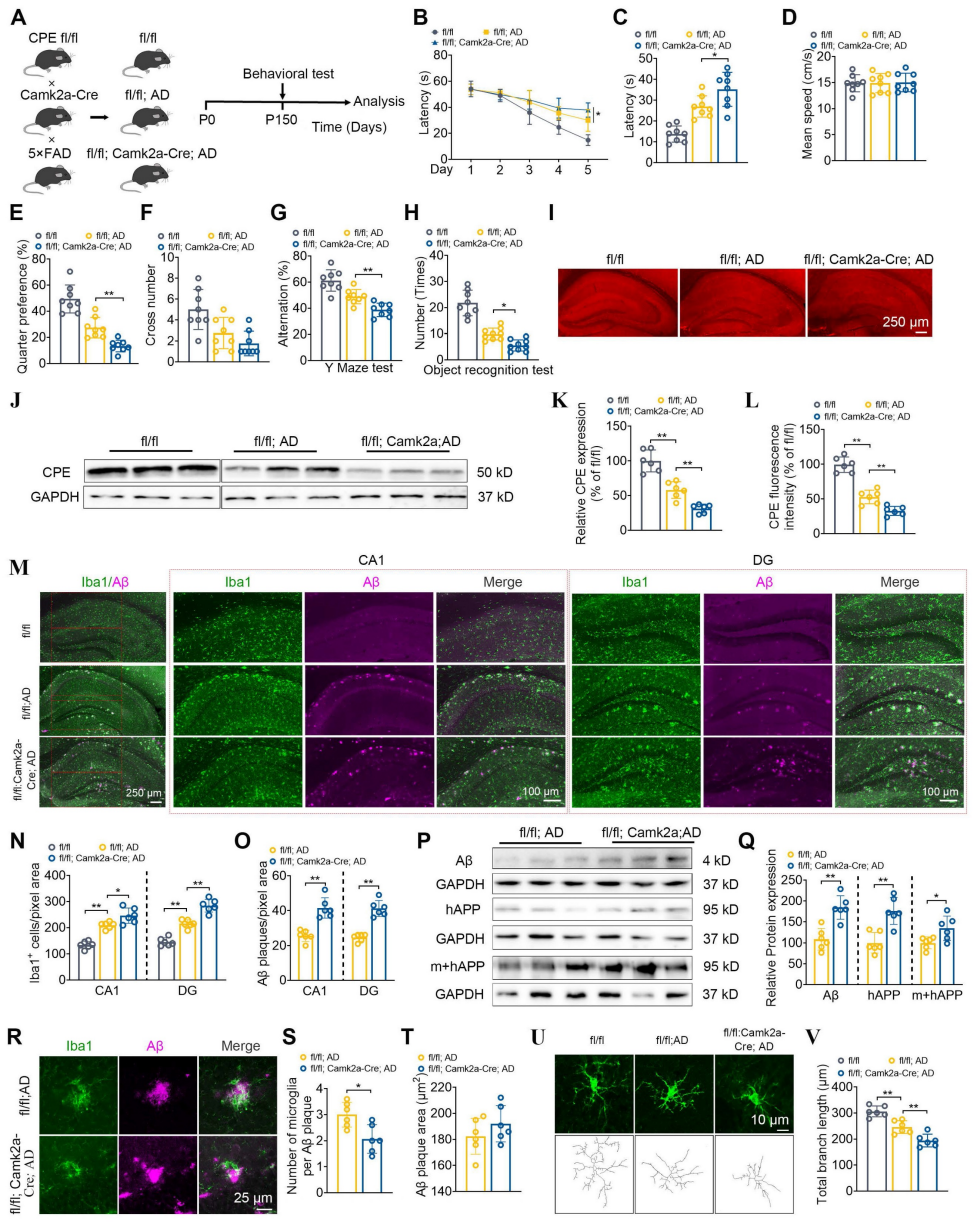
Neuronal CPE knockdown impairs cognitive functions, increases Aβ deposition, and elevates microglial numbers in 5×FAD mice at 5 months old. (A) Schematic illustrating the generation of fl/fl: Camk2a-Cre: AD mice, along with behavioral testing. P0, postnatal day 0. (B) Morris Water Maze (MWM) analysis measures the latency (s) to reach the target in the invisible platform training. (C) MWM analysis of Latency (s), n = 8 mice, One-way ANOVA analysis followed by Tukey's post-hoc multiple comparison test, [F (2,21) = 26.06, p < 0.0001; fl/fl: AD vs. fl/fl: Camk2a-Cre: AD, p = 0.0312]. (D) MWM analysis of Mean speed (centimeters per second), n = 8 mice, One-way ANOVA analysis followed by Tukey's post-hoc multiple comparison test, [F (2,21) = 0.0165, p = 0.9836]. (E) MWM Target quarter preference (%), n = 8 mice, One-way ANOVA analysis followed by Tukey's post-hoc multiple comparison test, [F (2,21) = 40.21, p < 0.0001; fl/fl: AD vs. fl/fl: Camk2a-Cre: AD, p = 0.0064]. (F) MWM Target cross number in the invisible platform tests, n = 8 mice, One-way ANOVA analysis followed by Tukey's post-hoc multiple comparison test, [F (2,21) = 9.127, p = 0.0014; fl/fl: AD vs. fl/fl: Camk2a-Cre: AD, p = 0.4198]. (G) Y maze test analysis as correct rate (%), n = 8 mice, One-way ANOVA analysis followed by Tukey's post-hoc multiple comparison test, [F (2,21) = 24.63, p < 0.0001; fl/fl: AD vs. fl/fl: Camk2a-Cre: AD, p = 0.0125]. (H) Object recognition test analysis as number (times), n = 8 mice, One-way ANOVA analysis followed by Tukey's post-hoc multiple comparison test, [F (2,23) = 55.21, p < 0.0001; fl/fl: AD vs. fl/fl: Camk2a-Cre: AD, p < 0.0254]. (I) Immunofluorescence was used to quantify the CPE relative fluorescence in the hippocampus of 5-month-old mice. (J) Western blot analysis reveals the levels of CPE in the hippocampus of 5-month-old mice. (K) CPE levels were normalized to GAPDH, n=6 mice, One-way ANOVA analysis followed by Tukey's post-hoc multiple comparison test, [F (2,15) = 49.84, p < 0.0001; fl/fl: AD vs. fl/fl: Camk2a-Cre: AD, p = 0.0064]. (L) Quantification of CPE relative fluorescence in the hippocampus of 5-month-old wild type and 5×FAD mice, n = 6 mice, average of 4-6 slices per mouse, average of 16 cells per slice, n = 6 mice, average of 4-6 slices per mouse, One-way ANOVA analysis followed by Tukey's post-hoc multiple comparison test, [F (2,15) = 79.98, p < 0.0001; fl/fl: AD vs. fl/fl: Camk2a-Cre: AD, p = 0.0054]. (M) Immunofluorescence was used to quantify the number of Iba1+ positive cells and Aβ plaque in the hippocampus of 5-month-old mice. (N and O) Quantification of Iba1+ positive cells and Aβ plaque in the CA1 and dentate gyrus, n = 6 mice, average of 4-6 slices per mouse, One-way ANOVA analysis followed by Tukey's post-hoc multiple comparison test, CA1, Iba1+: [F (2,15) = 47.69, p < 0.0001; fl/fl: AD vs. fl/fl: Camk2a-Cre: AD, p = 0.0154]; DG, Iba1+: [F (2,15) = 91.91, p < 0.0001; fl/fl: AD vs. fl/fl: Camk2a-Cre: AD, p < 0.0001]; CA1, Aβ: Unpaired t test, [p = 0.0002]; DG, Aβ: Unpaired t test, [p < 0.0001]. (P) Western blot analysis demonstrates the levels of Aβ, mouse APP and human+mouse APP in the hippocampus of 5-month-old fl/fl: AD and fl/fl: Camk2a-Cre: AD mice. (Q) Mouse APP and human+mouse APP levels were normalized to GAPDH. n=6 mice, Aβ: Unpaired t test, [p = 0.0006]; mouse APP: Unpaired t test, [p = 0.001]; human+mouse APP: Unpaired t test, [p = 0.024]. (R-T) Immunofluorescent staining of Iba1 and Aβ, along with the statistical analysis of microglia number per Aβ plaque and Aβ plaque size in fl/fl: AD and fl/fl: Camk2a-Cre: AD mice. n = 6 mice, average of 4-6 slices per mouse, microglia number: Unpaired t test, [p = 0.0101]; Aβ plaque size: Unpaired t test, [p = 0.2572]. (U and V) Immunofluorescent staining of Iba1 and the statistical analysis of total branch length (micrometers) in fl/fl: AD and fl/fl: Camk2a-Cre: AD mice. n = 6 mice, average of 4-6 slices per mouse, average of 16 cells per slice, One-way ANOVA analysis followed by Tukey's post-hoc multiple comparison test, [F (2,15) = 34.63, p < 0.0001; fl/fl: AD vs. fl/fl: Camk2a-Cre: AD, p = 0.0042].

Given that the expression of CPE in neurons of 5×FAD mice is reduced, we used a neuron-specific knockout of CPE mouse line using a Camk2a mouse and then crossed it with a 5×FAD mouse (Figure 2A). First, we compared 5 mth old (P150) fl/fl:AD mice with WT (fl/fl) mice and showed significantly impaired learning (Figure 2B) and memory (Figure 2C-F) in the Morris water maze test, Y maze (Figure 2G) and Novel Object Recognition (Figure 2H) tests which was correlated with the decreased NF-α1/CPE levels in the fl/fl:AD mice (Figure 2I-L). To investigate the role of CPE in the hippocampus of 5xFAD mice, we utilized Camk2a-Cre mice to delete the CPE gene in hippocampal neurons. The Camk2a promoter drives Cre expression in neurons but not in glial cells, allowing for neuron-specific expression knockdown 19. Our mouse construction involved crossing Camk2a-Cre mice with CPE fl/fl mice, which harbors loxP sites flanking the CPE gene (Cyagen Biosciences). This strategy resulted in the conditional deletion of CPE in hippocampal neurons upon Cre recombinase activity.

We then analyzed the hippocampus of 5 mth old 5xFAD fl/fl: Camk2a-Cre-AD mice which are 5xFAD fl/fl:AD mice that have NF-α1/CPE expression conditionally knocked down in brain neurons 19. These mice showed a further knock-down of NF-α1/CPE by ~50% compared to fl/fl:AD mice (Figure 2I-L). They also exhibited an increased deficit in learning (Figure 2B) and memory in the Morris water maze (Figure 2C-F), Y-maze (Figure 2G) and Novel Object Recognition (Figure 2H) tests compared with fl/fl:AD mice (Figure 2E-H). These observations indicate that lowering of NF-α1/CPE level in fl/fl: Camk2a-Cre-AD mice resulted in increased severity of memory loss. Next, we compared the number of Iba1positive microglia cells which reflect neuroinflammation state in the hippocampus of fl/fl-WT, fl/fl:AD and fl/fl: Camk2a-Cre-AD mice. Iba1 positive cells were greater in numbers in fl/fl: Camk2a-Cre-AD > fl/fl:AD> fl/fl-WT mice, in both the CA1 region and the dentate gyrus (Figure 2M, N). Analysis of Aβ plaques showed significantly greater numbers in fl/fl: Camk2a-Cre-AD versus fl/fl:AD mice (Figure 2M, O). Additionally, Aβ, human amyloid precursor protein (APP), and human +mouse APP levels were significantly higher in fl/fl: Camk2a-Cre-AD versus fl/fl:AD mice (Figure 2P, Q). Decreased NF-α1/CPE levels in fl/fl: Camk2a-Cre-AD mice resulted in greater numbers of microglia. However, when we investigated the number of microglia associated with each Aβ plaque, we found fewer numbers of microglia associated with each plaque in fl/fl: Camk2a-Cre-AD mice versus fl/fl:AD mice, no difference in Aβ plaque size (Figure 2R-T). Analysis of the morphology of the microglia in fl/fl mice revealed long branch length indicative of “resting state” compared with shorter branch length in fl/fl:AD mice and even fewer and shorter branch processes in fl/fl: Camk2a-Cre-AD mice characteristic of reactive microglia (Figure 2U, V).

We have also evaluated the cognitive function and AD pathology in the hippocampus of fl/fl: Camk2a-Cre-AD mice at 7 mths of age (Figure S2). NF-α1/CPE levels in these mice were decreased by ~50% compared to fl/fl:AD mice at the same age. Furthermore, they showed more severe cognitive function, increased Aβ deposition and expression and elevated microglial numbers compared to the fl/fl: AD mice of the same age (Figure S2A-O). The number of microglia per Aβ plaque and total branch length of the microglia were decreased in fl/fl: Camk2a-Cre-AD mice versus fl/fl:AD mice (Figure S2P-T). However, by 9 mths of age the fl/fl: Camk2a-Cre-AD mice did not show any further decrease of NF-α1/CPE level compared to fl/fl:AD mice at that age (Figure S3A-C). As suggested from the time course of decline of NF-α1/CPE with age in 5xFAD mice shown in Figure 1B, C, 9 mths old, fl/fl: AD mice and fl/fl: Camk2a-Cre-AD mice probably have reached maximum decline of NF-α1/CPE level. Consistent with no difference in NF-α1/CPE levels with fl/fl:AD mice, the fl/fl: Camk2a-Cre-AD mice at 9 mths showed no difference in cognitive dysfunction, Aβ deposition and expression, microglia numbers and their total branch length, as well as the number of microglia per Aβ plaque, compared to fl/fl:AD mice (Figure S3D-T).

### Neuronal NF-α1/CPE knock-down decreases synaptic proteins, dendritic branching and spine numbers in 5xFAD mice

**Figure 3:**
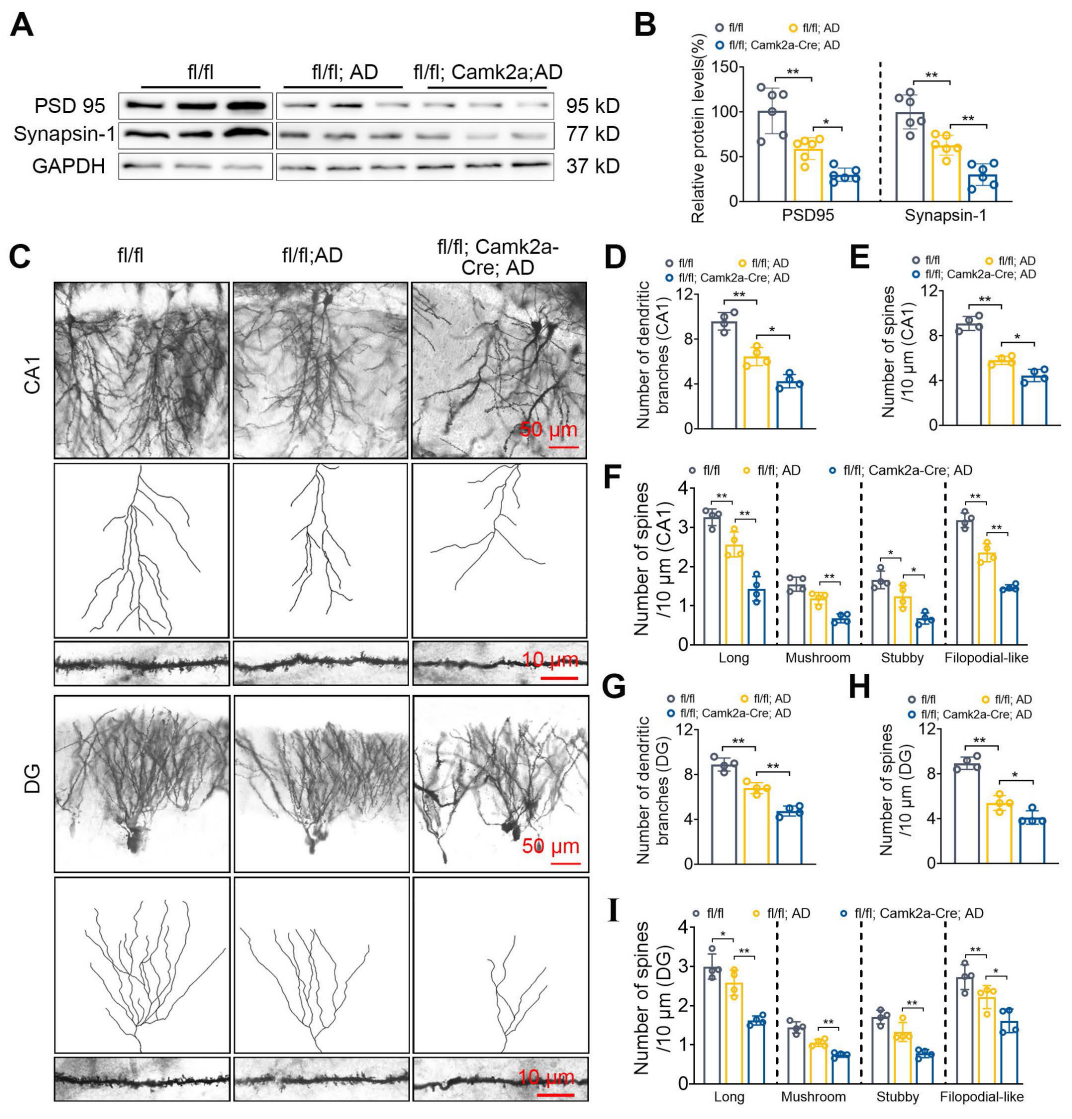
Neuronal CPE knockdown reduces synaptic proteins and modifies dendritic morphology in 5×FAD mice at 5 months old. (A) Western blot analysis reveals the levels of PSD 95 and Synapsin-1 in the hippocampus of 5-month-old fl/fl, fl/fl: AD, and fl/fl: Camk2a-Cre: AD mice. (B) PSD 95 and Synapsin-1 levels were normalized to GAPDH, n=6 mice, One-way ANOVA analysis followed by Tukey's post-hoc multiple comparison test, PSD 95: [F (2,15) = 27.05, p < 0.0001; fl/fl: AD vs. fl/fl: Camk2a-Cre: AD, p = 0.0236]; Synapsin-1: [F (2,15) = 35.18, p < 0.0001; fl/fl: AD vs. fl/fl: Camk2a-Cre: AD, p = 0.0036]. (C) Representative microscopy images of Golgi-Cox staining of CA1 and dentate gyrus. (D) Quantification of the number of dendritic branches in CA1. n = 4 mice, One-way ANOVA analysis followed by Tukey's post-hoc multiple comparison test, [F (2,9) = 83.40, p < 0.0001; fl/fl: AD vs. fl/fl: Camk2a-Cre: AD, p = 0.0056]. (E) Quantification of spine density in CA1. n = 4 mice, One-way ANOVA analysis followed by Tukey's post-hoc multiple comparison test, [F (2,9) = 53.56, p < 0.0001; fl/fl: AD vs. fl/fl: Camk2a-Cre: AD, p = 0.0134]. (F) Quantification of spine density (CA1) of long, n=4 mice, One-way ANOVA analysis followed by Tukey's post-hoc multiple comparison test, [F (2,9) = 42.04, p < 0.0001; fl/fl: AD vs. fl/fl: Camk2a-Cre: AD, p = 0.0008]; mushroom, One-way ANOVA analysis followed by Tukey's post-hoc multiple comparison test, [F (2,9) = 33.75, p < 0.0001; fl/fl: AD vs. fl/fl: Camk2a-Cre: AD, p = 0.0025]; stubby, One-way ANOVA analysis followed by Tukey's post-hoc multiple comparison test, [F (2,9) = 19.72, p = 0.0005; fl/fl: AD vs. fl/fl: Camk2a-Cre: AD, p = 0.0141]; filopodial-like, One-way ANOVA analysis followed by Tukey's post-hoc multiple comparison test, [F (2,9) = 94.32, p < 0.0001; fl/fl: AD vs. fl/fl: Camk2a-Cre: AD, p = 0.0001]. (G) Quantification of the number of dendritic branches in the dentate gyrus. n = 4 mice, One-way ANOVA analysis followed by Tukey's post-hoc multiple comparison test, [F (2,9) = 67.10, p < 0.0001; fl/fl: AD vs. fl/fl: Camk2a-Cre: AD, p = 0.0007]. (H) Quantification of spine density in the dentate gyrus. n = 4 mice, One-way ANOVA analysis followed by Tukey's post-hoc multiple comparison test, [F (2,9) = 69.39, p < 0.0001; fl/fl: AD vs. fl/fl: Camk2a-Cre: AD, p = 0.0334]. (I) Quantification of spine density (DG) of long, n=4 mice, One-way ANOVA analysis followed by Tukey's post-hoc multiple comparison test, [F (2,9) = 26.93, p = 0.0002; fl/fl: AD vs. fl/fl: Camk2a-Cre: AD, p = 0.0019]; mushroom, One-way ANOVA analysis followed by Tukey's post-hoc multiple comparison test, [F (2,9) = 43.77, p = 0.0067; fl/fl: AD vs. fl/fl: Camk2a-Cre: AD, p = 0.0056]; stubby, One-way ANOVA analysis followed by Tukey's post-hoc multiple comparison test, [F (2,9) = 26.42, p = 0.0002; fl/fl: AD vs. fl/fl: Camk2a-Cre: AD, p = 0.0054]; filopodial-like, One-way ANOVA analysis followed by Tukey's post-hoc multiple comparison test, [F (2,9) = 13.51, p = 0.0019; fl/fl: AD vs. fl/fl: Camk2a-Cre: AD, p = 0.0477].

The effect of decreased NF-α1/CPE levels in fl/fl: Camk2a-Cre-AD mice at 5 mths of age on synaptic proteins, PSD95 and synapsin-1 expression, as well as neuronal dendritic branches and dendritic spine numbers were analyzed in the hippocampus. Both PSD95 and synapsin1 expression were decreased in fl/fl: Camk2a-Cre-AD mice compared with fl/fl AD mice (Figure 3A, B). Additionally, the number of dendritic branches (Figure 3C, D) and spines (Figure 3E) were decreased in the CA1 neurons of fl/fl: Camk2a-Cre-AD mice compared with fl/fl AD mice. Analysis of different types of spines showed that the number of long, mushroom, stubby and filopodial-like spines were all decreased in fl/fl: Camk2a-Cre-AD mice versus fl/fl AD mice (Figure 3F). Likewise in the dentate gyrus, the number of dendritic branches (Figure 3C, G) and spines (Figure 3H) were decreased in fl/fl: Camk2a-Cre-AD mice compared with fl/fl AD mice. The different types of spines in the dentate gyrus show that the number of long, mushroom, stubby and filopodial-like spines were all decreased in fl/fl: Camk2a-Cre-AD mice compared with fl/fl AD mice (Figure 3I).

### Hippocampal delivery of AAV-CPE reverses cognitive dysfunction, amyloid deposition and microgliosis in 5xFAD mice

**Figure 4:**
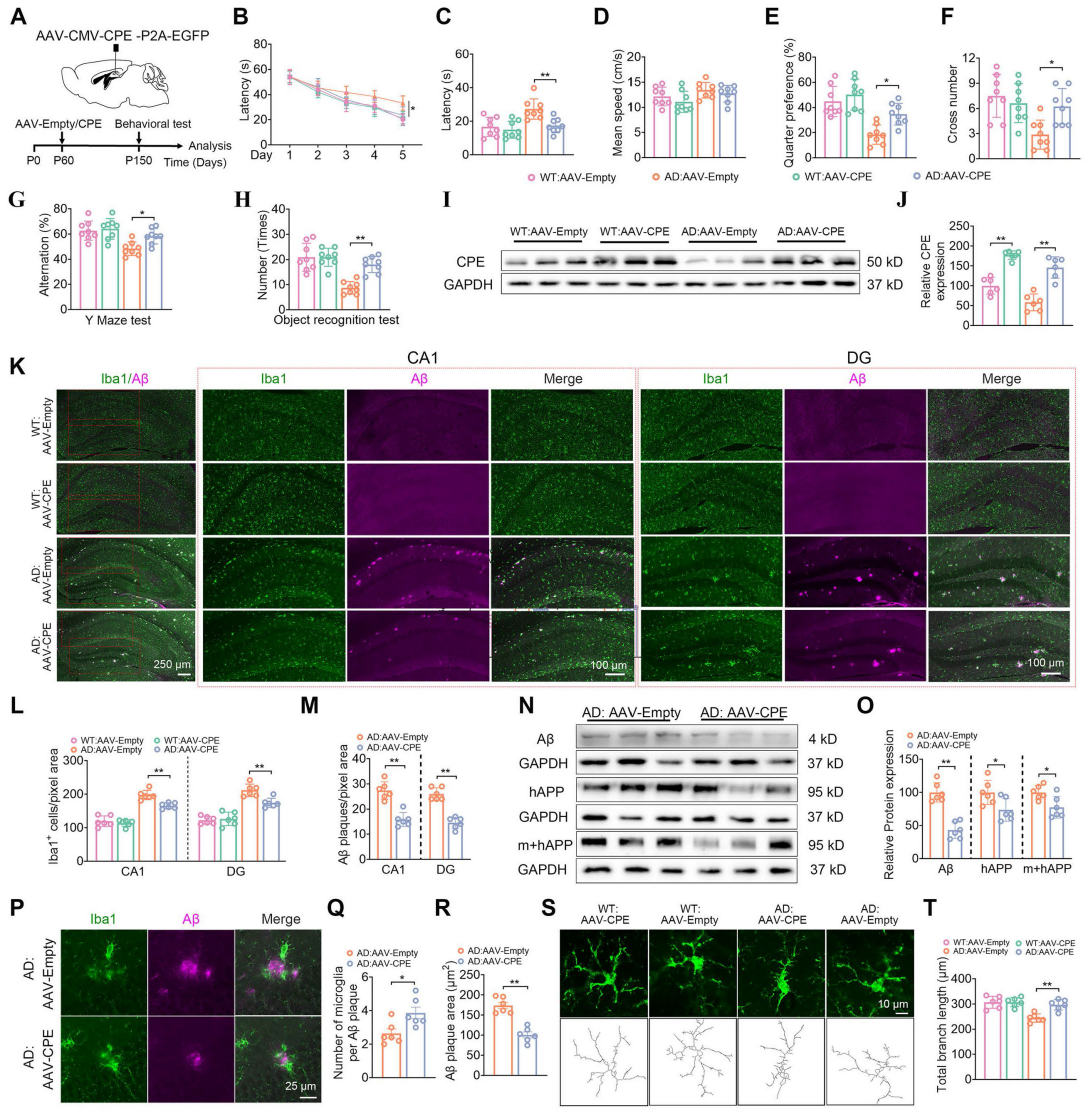
Enhanced CPE expression restores cognitive functions, reduces Aβ deposition, and diminishes microglial numbers in 5×FAD mice at 5 months old. (A) Schematic depicting the administration of AAV-Empty/AAV-CPE, along with behavioral assessments. P0, the day of birth. (B) Morris Water Maze (MWM) analysis measures the latency (s) to reach the target in the invisible platform training. (C) MWM analysis assesses latency (s), n = 8 mice, One-way ANOVA analysis followed by Tukey's post-hoc multiple comparison test, [F (3, 28) = 8.815, p = 0.0003; AD: AAV-Empty vs. AD: AAV-CPE, p = 0.0046]. (D) MWM: Mean speed (centimeters per second), n = 8 mice, One-way ANOVA analysis, [F (3, 28) = 2.331, p = 0.0957]. (E) MWM: Target quarter preference (%), n = 8 mice, One-way ANOVA analysis followed by Tukey's post-hoc multiple comparison test, [F (3, 28) = 14.51, p < 0.0001; AD: AAV-Empty vs. AD: AAV-CPE, p = 0.0239]. (F) MWM: Number of target crossings in the invisible platform tests, n = 8 mice, One-way ANOVA analysis followed by Tukey's post-hoc multiple comparison test, [F (3, 28) = 6.756, p = 0.0014; AD: AAV-Empty vs. AD: AAV-CPE, p = 0.0236]. (G) Y maze test analysis as the percentage of correct choices, n = 8 mice, One-way ANOVA analysis followed by Tukey's post-hoc multiple comparison test, [F (3, 28) = 8.339, p = 0.0004; AD: AAV-Empty vs. AD: AAV-CPE, p = 0.0379]. (H) Object recognition test analysis as the number of recognized objects, n = 8 mice, One-way ANOVA analysis followed by Tukey's post-hoc multiple comparison test, [F (3, 28) = 17.47, p < 0.0001; AD: AAV-Empty vs. AD: AAV-CPE, p = 0.0003]. (I) Western blot analysis showcases the levels of CPE in the hippocampus of 5-month-old mice (each group, n = 6). (J) CPE levels were normalized to GAPDH, n = 6 mice, One-way ANOVA analysis followed by Tukey's post-hoc multiple comparison test, [F (3,20) = 37.63, p < 0.0001; WT: AAV-Empty vs. WT: AAV-CPE, p < 0.0001; AD: AAV-Empty vs. AD: AAV-CPE, p < 0.0001]. (K) Immunofluorescence was used to quantify the number of Iba1+ positive cells and Aβ plaques in the hippocampus of 5-month-old mice. (L and M) Quantification of Iba1+ positive cells and Aβ plaques in the CA1 and dentate gyrus, n = 6 mice, averaged from 4-6 slices per mouse, One-way ANOVA analysis followed by Tukey's post-hoc multiple comparison test, Iba1+: [F (3,20) = 61.09, p < 0.0001; AD: AAV-Empty vs. AD: AAV-CPE, p = 0.0014]; DG, Iba1+: [F (3, 20) = 40.76, p < 0.0001; AD: AAV-Empty vs. AD: AAV-CPE, p = 0.0035]; CA1, Aβ: Unpaired t test, [p < 0.0001]; DG, Aβ: Unpaired t test, [p < 0.0001]. (N) Western blot analysis demonstrates the levels of Aβ, hAPP, m+hAPP in the hippocampus of 5-month-old 5×FAD mice treated with AAV-Empty or AAV-CPE, n = 6 mice. (O) Aβ, hAPP, m+hAPP levels were normalized to GAPDH, n = 6 mice, Unpaired t test, [ Aβ: p < 0.0001; hAPP: p = 0.0276; m+hAPP: p = 0.0178]. (P-R) Immunofluorescent staining of Iba1 and Aβ, along with the statistical analysis of the number of microglia per Aβ plaque and Aβ plaque size in 5×FAD mice treated with AAV-Empty or AAV-CPE, n = 5 mice, average of 4-6 slices per mouse, microglia number: Unpaired t test, [p = 0.0184]; Aβ plaque size: Unpaired t test, [p < 0.0001]. (S and T) Immunofluorescent staining of Iba1, along with the statistical analysis of total branch length (micrometers) in WT:AAV-CPE, WT:AAV-Empty, AD:AAV-CPE and AD:AAV-Empty mice, n = 6 mice, average of 4-6 slices per mouse, average of 16 cells per slice, One-way ANOVA analysis followed by Tukey's post-hoc multiple comparison test, [F (3,28) = 14.51, p < 0.0001; AD:AAV-Empty vs. AD:AAV-CPE, p = 0.0011].

Given that the knock down of NF-α1/CPE level in fl/fl: Camk2a-Cre-AD mice resulted in increased severity of AD pathology, we hypothesized that increasing CPE expression might reverse cognitive dysfunction and AD pathology in 5xFAD mice. We injected AAV-CMV-CPE-P2A-EGFP (AAV-CPE) or AAV-empty vector in the hippocampus bilaterally in 5xFAD and WT mice at P60 (2 mths) of age and assayed them at P150 (5 mths) of age (Figure 4A). Delivery of AAV-CPE in the hippocampus increased NF-α1/CPE expression ~2-3 fold in WT and AD mice and reversed spatial learning and memory (Figure 4B-J) deficits as shown in the Morris water maze test in the AD mice. Likewise, AAV-CPE treatment rescued spatial working and recognition memory in the Y maze (Figure 4G) and Novel Object Recognition tests (Figure 4H), respectively, in AD mice. WT mice treated with AAV-CPE showed no change in the different cognitive tests.

Analysis of Iba1 and GFAP positive stained microglia cells in the CA1 region and the DG revealed higher numbers in the AD mice relative to WT mice, which was attenuated in the AAV-CPE treated AD mice (Figure 4K, L, Figure S4). Additionally, the number of Aβ plaques decreased in the CA1 region and DG in AD mice after AAV-CPE delivery (Figure 4K, M). Expression of Aβ peptide and its precursor protein APP were also reduced in the AAV-CPE treated AD mice versus untreated (AAV-Empty) AD mice (Figure 4N, O). Interestingly when we analyzed the number of microglia associated with plaques, we found that there were more microglia per plaque and decreasrd Aβ plaques in AAV-CPE treated mice versus untreated AD mice (Figure 4P-R). Comparison of the morphology of the microglia cells (Figure 4S, T) showed that total branching length of the cells in AAV-CPE treated mice was similar to WT mice; whereas, untreated AD mice showed microglia cells with less total branching length, characteristic of reactive microglia.

AD mice after hippocampal delivery of AAV-CPE at 2 mths (P60) of age were also evaluated at 7mths (P210) (Figure S5) and 9 mths (P270) (Figure S6). At 7mths of age, the AAV-CPE treated mice showed ~2-fold increase in CPE expression and learning and memory deficits evident in untreated AD mice were reversed in these mice (Figure S7A-J). Furthermore, the number of Iba1 positive stained microglia cells and GFAP positive stained astroglia cells in the CA1 region and the DG in AAV-CPE treated AD mice were reduced compared to untreated AD mice (Figure S5K, L, Figure S6). Additionally, the number of Aβ plaques in the CA1 region and DG in the AD mice after AAV-CPE treatment (Figure S5K, M) and expression of Aβ peptide (Figure S5N, O) was reduced in the AAV-CPE treated versus untreated AD mice. We also found that the number of microglia associated with each plaque were greater and Aβ plaques were decreased in AAV-CPE treated mice versus untreated AD mice (Figure S5P-R). Moreover, the morphology of the microglia cells (Figure S5S, T) showed that total branching length in AAV-CPE treated mice was greater than in untreated AD mice. Analysis of 9 mth old AD mice after hippocampal AAV-CPE delivery showed an ~2-fold increase in CPE expression compared to untreated (AAV-empty) AD mice (Figure S7A-C). Results similar to 5 and 7 mth old AD mice in the rescue of learning and memory deficits (Figure S7D-J) and reduction of Iba1^+^ microglia and GFAP ^+^ astroglia cell number (Figure S7K, L, Figure S8) and Aβ plaques and expression (Figure S7M-O) were obtained for the AAV-CPE treated versus untreated AD mice. Likewise, an increase in the number of microglia per plaque and total branch length and decreased Aβ plaques were observed in AAV-CPE treated compared to untreated AD mice (Figure S7P-T).

### Hippocampal delivery of AA-CPE rescues synaptic protein expression, dendritic branching and spine numbers in 5xFAD mice

**Figure 5:**
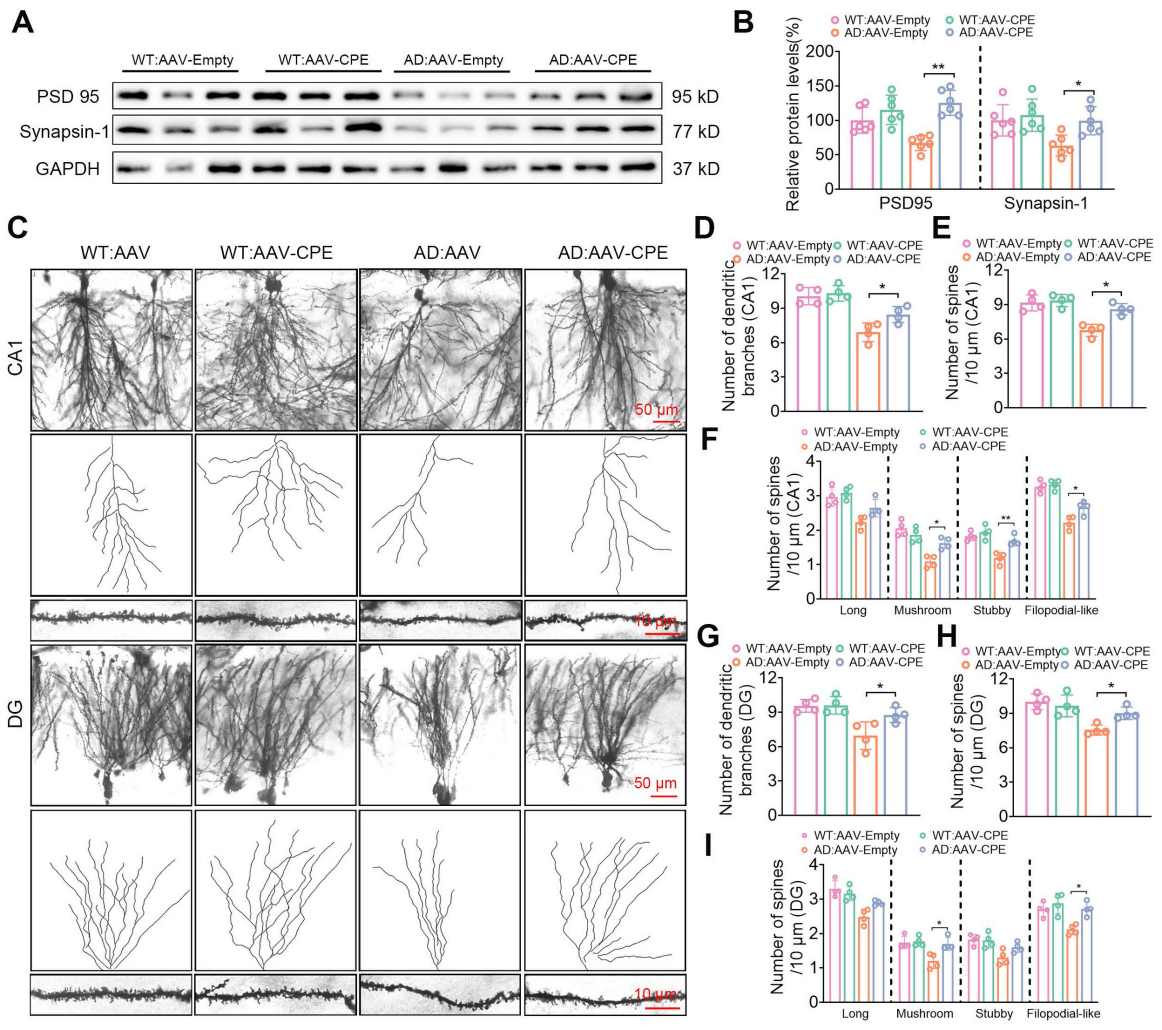
Enhanced CPE expression restores synaptic protein levels and dendritic morphology in 5×FAD mice at 5 months old. (A) Western blot analysis showcases the levels of PSD 95 and Synapsin-1 in the hippocampus of 5-month-old 5×FAD mice treated with AAV-Empty or AAV-CPE. (B) PSD 95 and Synapsin-1 levels were normalized to GAPDH. n=6 mice, One-way ANOVA analysis followed by Tukey's post-hoc multiple comparison test, PSD 95: [F (3,20) = 12.42, p < 0.0001; AD: AAV-Empty vs. AD: AAV-CPE, p < 0.0001]; Synapsin-1: [F (3,20) = 5.453, p = 0.0066; AD: AAV-Empty vs. AD: AAV-CPE, p = 0.030]. (C) Representative microscopy images of Golgi-Cox staining in the CA1 and dentate gyrus. (D) Quantification of the number of dendritic branches in the CA1. n = 4 mice, One-way ANOVA analysis followed by Tukey's post-hoc multiple comparison test, [F (3,12) = 18.31, p < 0.0001; AD: AAV-Empty vs. AD: AAV-CPE, p = 0.050]. (E) Quantification of spine density in the CA1. n = 4 mice, One-way ANOVA analysis followed by Tukey's post-hoc multiple comparison test, [F (3,12) = 17.50, p < 0.0001; AD: AAV-Empty vs. AD: AAV-CPE, p = 0.0386]. (F) Quantification of spine density (CA1) of long, n=4 mice, One-way ANOVA analysis followed by Tukey's post-hoc multiple comparison test, [F (3,12) = 11.21, p = 0.0009; AD: AAV-Empty vs. AD: AAV-CPE, p = 0.1022]; mushroom, One-way ANOVA analysis followed by Tukey's post-hoc multiple comparison test, [F (3,12) = 17.78, p = 0.0001; AD: AAV-Empty vs. AD: AAV-CPE, p = 0.0107]; stubby, One-way ANOVA analysis followed by Tukey's post-hoc multiple comparison test, [F (3,12) = 16.20, p = 0.0002; AD: AAV-Empty vs. AD: AAV-CPE, p = 0.0043]; filopodial-like, One-way ANOVA analysis followed by Tukey's post-hoc multiple comparison test, [F (3,12) = 34.95, p < 0.0001; AD: AAV-Empty vs. AD: AAV-CPE, p = 0.0190]. (G) Quantification of the number of dendritic branches in the dentate gyrus. n = 4 mice, One-way ANOVA analysis followed by Tukey's post-hoc multiple comparison test, [F (3,12) = 8.830, p = 0.0023; AD: AAV-Empty vs. AD: AAV-CPE, p = 0.0431]. (H) Quantification of spine density in the dentate gyrus. n = 4 mice, One-way ANOVA analysis followed by Tukey's post-hoc multiple comparison test, [F (3,12) = 10.08, p = 0.0013; AD: AAV-Empty vs. AD: AAV-CPE, p = 0.0468]. (I) Quantification of spine density (DG) of long, n = 4 mice, One-way ANOVA analysis followed by Tukey's post-hoc multiple comparison test, [F (3,12) = 12.80, p = 0.0005; AD: AAV-Empty vs. AD: AAV-CPE, p = 0.1022]; mushroom, One-way ANOVA analysis followed by Tukey's post-hoc multiple comparison test, [F (3,12) = 8.459, p = 0.0027; AD: AAV-Empty vs. AD: AAV-CPE, p = 0.0112]; stubby, One-way ANOVA analysis followed by Tukey's post-hoc multiple comparison test, [F (3,12) = 6.670, p = 0.0067; AD: AAV-Empty vs. AD: AAV-CPE, p = 0.1673]; filopodial-like, One-way ANOVA analysis followed by Tukey's post-hoc multiple comparison test, [F (3,12) = 9.092, p = 0.0020; AD: AAV-Empty vs. AD: AAV-CPE, p = 0.0109].

The hippocampal delivery of AAV-CPE in AD mice also had an effect on restoring expression of synaptic proteins, PSD 95 and synapsin-1, which were decreased in AD mice, to levels comparable to WT mice (Figure 5A, B). Additionally, the number of dendritic branches and spines in the CA1 (Figure 5C-E) and DG (Figure 5C, G, H) neurons of AD mice treated with AAV-CPE were restored to levels similar to WT mice. Analysis of different types of spines in CA1 (Figure 5F) and DG (Figure 5I) neurons showed that the number of long spines were similar between WT, AD and AAV-CPE treated mice. However, the mushroom, stubby and filopodial-like spines were decreased in AD versus WT mice, but the numbers were restored with AAV-CPE treatment, although the increase in stubby spines in the DG neurons did not reach significance.

### Overexpression of CPE in the hippocampus reduces Aβ p

### laque load in a dose-dependent manner in 5xFAD mice

**Figure 6:**
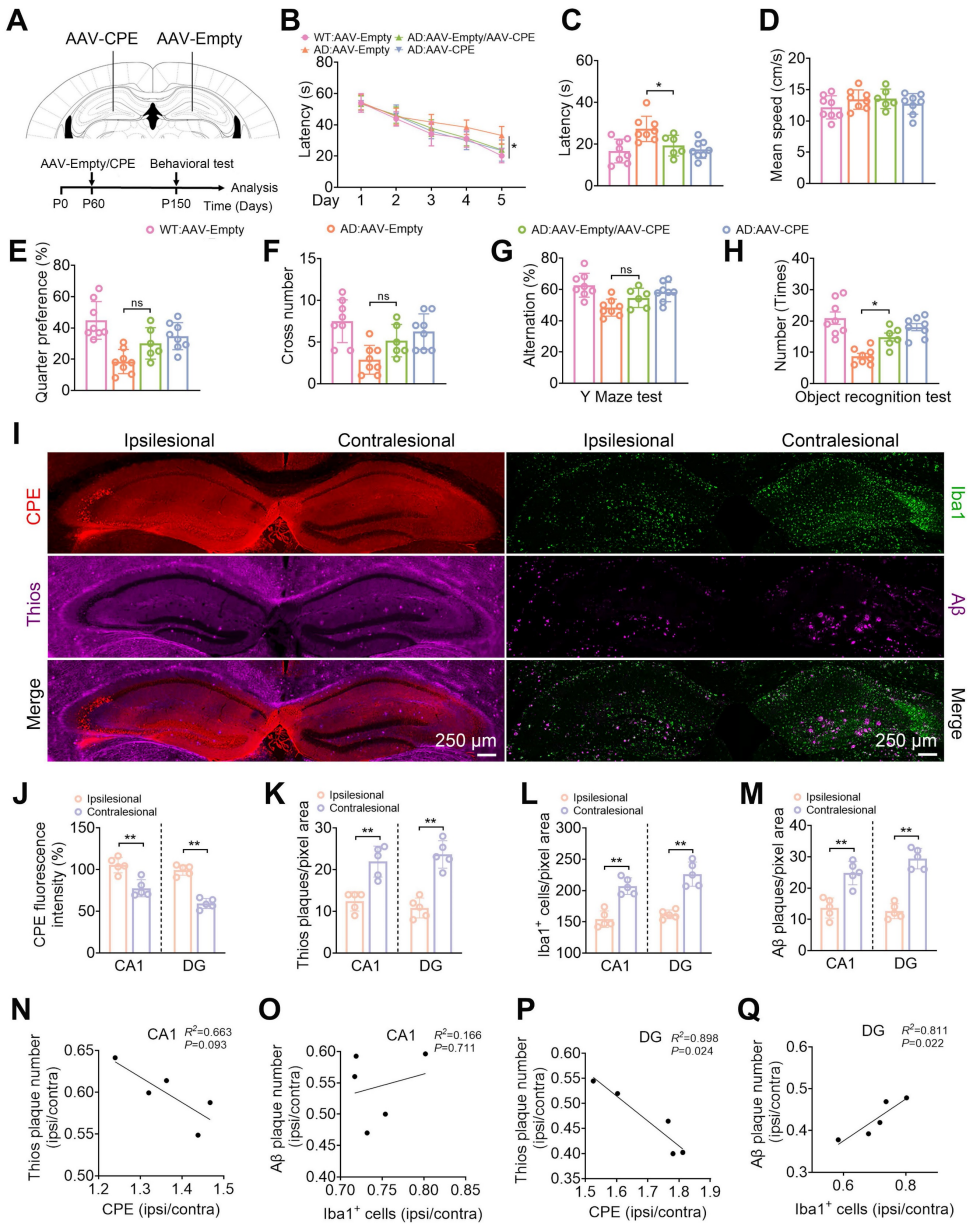
CPE overexpression in the hippocampus reduces Aβ plaque load in 5xFAD mice. (A) Schematic depicting the injection of Ipsilesional-AAV-CPE and Contralseional-AAV-Empty, along with behavioral assessments. P0, the day of birth. (B) Morris Water Maze analysis determines the latency (s) to reach the invisible platform during training. Morris Water Maze analysis assesses (C) latency (s), n = 6-8 mice, One-way ANOVA analysis followed by Tukey's post-hoc multiple comparison test, [F (3,26) = 6.756, p = 0.0016; AD: AAV-Empty vs. AD: AAV-Empty/ AAV-CPE, p = 0.0438]. (D) mean speed (centimeters per second), n = 6-8 mice, One-way ANOVA analysis followed by Tukey's post-hoc multiple comparison test, [F (3,26) = 1.078, p = 0.3756]. (E) target quarter preference (%), n = 6-8 mice, One-way ANOVA analysis followed by Tukey's post-hoc multiple comparison test, [F (3,26) = 10.06, p = 0.0001; AD: AAV-Empty vs. AD: AAV-Empty/ AAV-CPE, p = 0.1474]. (F) number of target crossings in the invisible platform tests, n = 6-8 mice, One-way ANOVA analysis followed by Tukey's post-hoc multiple comparison test, [F (3,26) = 6.822, p = 0.0015; AD: AAV-Empty vs. AD: AAV-Empty/ AAV-CPE, p = 0.2144]. (G) Y maze test analysis as the percentage of correct choices, n = 6-8 mice, One-way ANOVA analysis followed by Tukey's post-hoc multiple comparison test, [F (3,26) = 7.131, p = 0.0012; AD: AAV-Empty vs. AD: AAV-Empty/ AAV-CPE, p = 0.2925]. (H) Object recognition test analysis as the number of recognized objects, n = 6-8 mice, One-way ANOVA analysis followed by Tukey's post-hoc multiple comparison test, [F (3,26) = 14.64, p < 0.0001; AD: AAV-Empty vs. AD: AAV-Empty/ AAV-CPE, p = 0.0328]. (I) Representative images of the contralateral and ipsilateral hemispheres of AAV-Empty and AAV-CPE-injected 5xFAD mice. Quantification of (J) CPE fluorescence intensity, n = 5 mice, averaged from 4-6 slices per mouse, Unpaired t test, [CA1, p = 0.0019; DG: p = 0.0001]. (K) Thioflavin S plaque, n = 5 mice, averaged from 4-6 slices per mouse, Unpaired t test, [CA1, p = 0.0011; DG: p = 0.0001]. (L) Iba1+ cell, n = 5 mice, averaged from 4-6 slices per mouse, Unpaired t test, [CA1, p = 0.0002; DG: p = 0.0001]. (M) Aβ plaque immunostaining, n = 5 mice, averaged from 4-6 slices per mouse, Unpaired t test, [CA1, p = 0.0010; DG: p < 0.0001]. (N-Q) Pearson correlation analyses of CPE levels (normalized to the contralateral side) with the number of ThioS+, Iba1+ cells (normalized to the contralateral side) with the number of Aβ plaques in the CA1 and dentate gyrus, n = 5 mice, averaged from 4-6 slices per mouse.

We further modified NF-α1/CPE expression in the hippocampus of 5xFAD mice by carrying out AAV-CPE injection on ipsilateral side, and AAV-empty vector on the contralateral side, in 5 mth old mice (Figure 6A). This level of NF-α1/CPE overexpression was able to rescue learning and memory deficits in these AD mice, to that approaching AD mice that received bilateral injection of AAV-CPE, as assayed by the Morris Water Maze, Y-Maze and Novel Object Recognition tests (Figure 6B-H). Expression levels of NF-α1/CPE in the ipsilesional relative to the contralesional side was ~1.37-fold and ~ 1.57-fold in the CA1 region and DG, respectively (Figure 6i, j). Additionally, Thioflavin-S and Aβ plaques (Figure 6I, K, M), as well as Iba1 positive microglia cells (Figure 6I, L) were decreased in the CA1 and DG areas on the ipsilesional side of the hippocampus treated with AAV-CPE compared to the (AAV-empty injected) contralesional side. Analysis of the relative amounts of NF-α1/CPE (ipsilateral/contralateral) versus Thio-S positive plaques (ipsilateral/contralateral) showed a significant inverse correlation in a dose -dependent manner in both the CA1 and DG region (Figure 6N, P). Interestingly, the relative amounts of Iba1^+^ microglia cells (ipsilateral/contralateral) versus Aβ plaques (ipsilateral/contralateral) showed a significant positive correlation in a dose -dependent manner in the DG, (Figure 6Q) although while it trended in the same manner in the CA1 region, was not statistically significant (Figure 6O).

## Discussion

Deciphering key players that control AD progression is critical to facilitate developing treatment strategies and therapeutic agents for the disease. In this study we have investigated the role of NF-α1/CPE in modulating the pathogenesis of AD. We utilized multiple paradigms to modify NF-α1/CPE expression levels, as well as physiological depletion during aging to demonstrate a direct correlation between the levels of NF-α1/CPE and deficit in cognitive function and disease pathology in 5xFAD mice. Our studies showed that during aging there is progressive decrease of NF-α1/CPE protein levels in both WT and 5xFAD mice, with the latter showing a faster rate of depletion (Figure 1). However, these observations are based on NF-α1/CPE level assessments at the age of 8 months, and as such, they may not fully capture the entire trajectory of NF-α1/CPE expression changes over the aging process. Moreover, this decrease was in neurons and not microglia or astroglia. We found that at 5 mths of age, there was a 75% and 54% decrease in NF-α1/CPE mRNA and protein respectively in AD (fl/fl:AD) mice compared to WT mice at the same age. This is correlated with a significant decline in learning and memory and increase in microgliosis in the AD mice. Further suppression of NF-α1/CPE expression in the fl/fl:AD mice by conditional knock down in neurons resulted in another ~50% decrease in NF-α1/CPE level in the fl/fl: Camk2a-Cre-AD mice. This led to a further decline in learning and memory, enhanced microgliosis with changes in microglia morphology characteristic of reactive microglia, and an increase in APP biosynthesis and Aβ plaques in these fl/fl: Camk2a-Cre-AD mice, compared to fl/fl:AD mice (Figure 2). Down regulation of expression of synaptic proteins and diminishing numbers of dendritic branching and spines were also observed which was more severe in fl/fl: Camk2a-Cre-AD > fl/fl:AD > WT mice (Figure 3). These changes likely lead to impairment of synaptic function. Based on our results showing that CPE, PSD95, and Synapsin-1 are all reduced to approximately 30%, we speculate that the decrease in CPE is correlated with the reduction of synaptic proteins. The observation that CPE is specifically diminished in neurons and not in microglia or astrocytes in the AD model mice further supports this correlation. Studies on 7 mths old fl/fl: Camk2a-Cre-AD mice which showed a further decrease in NF-α1/CPE expression compared to fl/fl;AD mice at the same age demonstrated a progressive decline in cognitive function, enhanced microgliosis and increase in Aβ plaques (Figure S2). By 9 mths of age, there was no further decline in NF-α1/CPE level in fl/fl: Camk2a-Cre-AD mice compared to fl/fl:AD mice of the same age. No change in cognitive function or AD pathology was observed between the two genotypes at this age (Figure S3). Thus, these results indicate there is a close correlation between decrease in NF-α1/CPE levels modified by genomic manipulation or physiological aging and severity of AD pathology, in a dose-dependent manner in AD mice. To date there has not been any studies done to knock down NF-α1/CPE systematically during aging to demonstrate its importance in controlling cognitive function and pathology in AD mice.

Overexpression of NF-α1/CPE in the hippocampus through delivery of AAV-CPE also indicated a correlation between increase in NF-α1/CPE levels and reversal of AD pathology in 5 x FAD mice. Bilateral injection of CPE-AAV in the hippocampus of 5 x FAD mice at 2 mths of age increased NF-α1/CPE level by ~2-3 fold and led to complete reversal of learning and memory loss, and reduced APP and Aβ expression and plaques, and microgliosis, assayed at age 5, 7 and 9 mths (Figure 4, S4, S5). Moreover, AAV-CPE treatment rescued the expression of synaptic proteins and reversed the loss of neuronal dendritic branching and spine numbers. The enhanced number of microglia per plaque and total branch length of microglia to “resting state” morphology were also restored. The increased number of microglia associated per plaque might indicate a role of these cells in the clearance of Aβ plaques. Previous studies have found that microglia surrounding plaques have a role in plaque clearance 24,30,31. Our findings indicate that overexpression of CPE can increase the number of microglia around the plaques, which is conducive to plaque removal. Conversely, a reduction in neuronal CPE expression leads to a decrease in microglia surrounding the plaques, suggesting that CPE may modulate microglial function.

The present study provides the evidence supporting the role of CPE in the pathophysiology of AD. Recent studies have demonstrated the therapeutic potential of enhancing CPE expression in the hippocampus as a strategy to combat the neuropathological features of AD. For instance, a study reported that increasing CPE expression through gene delivery in the 3xTg-AD mouse model successfully rescued neurodegeneration, cognitive dysfunction, and tau hyperphosphorylation 22. This finding suggests that modulating CPE expression may have a profound impact on the progression of AD. Similarly, another study has shown that delivering microRNA agomirs, which can increase CPE expression, via intracerebroventricular injection or intranasal instillation in the APP/PS1 mouse model also led to significant improvements in the same neuropathological hallmarks of AD 32. These interventions were associated with a reduction in amyloid plaques, improved cognitive performance, and decreased neuroinflammation, as indicated by reduced microglia activation. Our findings using 5xFAD mouse model align with these previous studies, further confirming the neuroprotective effects of enhancing CPE expression. In our study, we observed decreased amyloid plaque accumulation, mitigated spatial memory deficits, and normalized hippocampal synaptogenesis and microglial anomalies across early and late stages of the disease, similar to the rescue of neurodegenerative processes seen in the 3xTg-AD and APP/PS1 mouse models after CPE augmentation. The consistency of these findings across different models and methodologies underscores the robustness of the approach and suggests that CPE expression modulation could be a viable therapeutic target for AD.

To further establish a dose-dependent correlation between NF-α1/CPE level and amyloid load, we injected AAV-CPE in one side (ipsilesional) and AAV-empty vector on the other side (contralesional) of the hippocampus of AD mice. Assay of these mice revealed a lower increase of NF-α1/CPE expression in the hippocampus than bilateral injection, but was sufficient to reverse cognitive dysfunction comparable to AAV-CPE bilaterally injected mice. Moreover, comparison of the ipsilesional and contralesional ratios of CPE and Thio-s plaques demonstrated an inverse relationship between hippocampal NF-α1/CPE levels and Aβ plaque load in a dose-dependent manner. This is consistent with previous studies showing that NF-α1/CPE overexpression in 3xTg-AD mice can down regulate the expression of APP transcriptionally and translationally, and reduce the production of insoluble Aβ1-42 in the hippocampus 22 Moreover, the positive relationship and the ability of NF-α1/CPE to prevent and reverse spatial memory deficits, normalize hippocampal synaptogenesis and microglial cell anomalies in pre- 22 and post-symptomatic mice reported here for different AD mouse models, emphasizes its potential in preventing and treatment of AD. Its mechanisms of action regulating multi-metabolic targets 22 such as inducing pro-survival and mitophagy while suppressing inflammation, renders it an efficacious therapeutic agent. Future studies will explore non-invasive procedures to deliver NF-α1/CPE to the brain, for example, through use of extracellular vesicle carriers, administered nasally 33.

In conclusion, using multiple paradigms to modulate the level of expression of NF-α1/CPE, in “loss” and “gain” of function studies, we have established a direct dose-dependent relationship between NF-α1/CPE levels in the hippocampus and AD pathology and cognitive function in 5xFAD mice, supporting a causal effect. These studies indicate that NF-α1/CPE is a key player in the control of AD pathogenesis. Hence, NF-α1/CPE is a potentially excellent therapeutic agent for the prevention and reversal of AD progression.

## Supplementary Material

Supplementary materials and methods, figures.

## Funding

This study was supported by the National Natural Science Foundation of China (No. 82071676).
