# Supplementary material for: Neurotrophic factor-α1/carboxypeptidase E controls progression and reversal of Alzheimer's disease pathogenesis in mice
Source: Theranostics. 2025 Jan 13;15(6):2279–92. doi: 10.7150/thno.99908 (PMC11840748; doi:10.7150/thno.99908)
Supplement: Supplementary file 1 — Supplementary materials and methods, figures. [file thnov15p2279s1.pdf]

SUPPORTING INFORMATION

MATERIALS AND METHODS

Genotyping of animals

Mouse samples of toes were collected at two months old age for isolating DNA for PCR analysis, which would aid in the identification of the genotype. The sequences of the primers used are as follows:

Flox-Forward primer: 5'-CTAAAGACACTGCATCCCTCTCTG-3',

Flox-Reverse primer: 5'-ATGTAAGCCCACATATTGTCTCTGT-3';

Cre-Forward primer: 5'-CATATTGGCAGAACGAAAACGC-3',

Cre-Reverse primer: 5'-CCTGTTTCACTATCCAGGTTACGG-3';

CPE-Forward primer: 5'-CTAAAGACACTGCATCCCTCTCTG-3',

CPE-Reverse primer: 5'-ATGTAAGCCCACATATTGTCTCTGT-3'.

The DNA underwent an initial denaturation step at 94 °C for 3 min, followed by denaturation at 94 °C for 30 s. Subsequently, the annealing step was performed at 62 °C for 30 s, followed by extension at 72 °C for 35 s. The final extension was carried out at 72 °C for 5 min. The products generated were then analyzed by 2% agarose gel electrophoresis and the results were evaluated using the Gel Image System (Tanon, Shanghai, China).

Behavioral tests

Morris Water Maze test

Mice were trained per day for 5 days with the platform hidden, spatial cues presented, and starting in a new quadrant for each trial. If they failed to locate the hidden platform, they were guided to it and granted 30 s to sit on the platform. On the 6th day of testing, the hidden platform was removed, and the velocity, time taken to cross the platform for the first time, quarter preference, and cross platform number were recorded.

Y maze

The Y-maze experiment involved positioning mice within the center of a Y-shaped maze (arm dimensions: 30 cm in length, 6 cm in width; wall height: 15 cm). The three arms were respectively labeled A, B, and C, with entries into each arm being recorded. For a duration of 7 min, the number and changes in entries into the arms were monitored using

a video-imaging system (Taimeng, Chengdu, China). The outcome was computed as the ratio of correct changes to total arm entries.

Novel object recognition test

In order to assess recognition, mice were placed in an apparatus (dimensions: 50 cm × 50 cm × 40 cm) one day prior to the test for habituation. On day 1 of the testing, the mice were permitted free exploration for 10 min, with two identical objects positioned on the diagonal of the apparatus. On day 2, one novel object was introduced in place of the other on the same diagonal as day 1. The animals were allowed free exploration for 7 min. The time of exploration within 2-3 cm around the novel object was recorded for the last 5 min.

Quantitative RT-PCR

Total RNA was isolated from brain tissues using TRIzol reagent. One microgram of RNA was utilized in a one-step first-strand cDNA synthesis kit (Genstar Biotech, Beijing, China). Quantitative real-time (RT) polymerase chain reaction (PCR) was conducted using 2× SYBR Green PCR master mix (Genstar Biotech). The primers utilized for analysis are listed as follows:

GAPDH-F: 5'-GCAAGTTCAACGGCACAG-3',

GAPDH-R: 5'-GCCAGTAGACTCCACGACAT-3';

CPE-F: 5'-CAGTGGTGGATCTTGAGGTCATTGC-3',

CPE-R: 5'-TTCTGCCATCATACGCTCTGTTGTC-3'. The mRNA level of CPE was normalized to the expression levels of GAPDH.

Immunofluorescence stain

Animals were perfused with normal saline, and the brains were fixed with 4% paraformaldehyde overnight at 4 °C. Subsequently, the brains were cryoprotected in 30% sucrose and subjected to frozen sectioning. The brains were sectioned coronally at 30 μm and stained with mouse anti-CPE antibody (1:1000; BD Bioscience, New Jersey, USA), rabbit anti-Iba1 antibody (1:1000; Wako, Osaka, Japan), rabbit anti- NeuN (1:1000, Cell Signaling Technology, Boston, MA, USA) and Aβ antibody (1:500; Covance, New Jersey, USA). The secondary antibodies employed were Alexa Fluor 594 goat anti-mouse secondary antibody (1:1000; Invitrogen, Carlsbad, CA) or Alexa Fluor 594 goat anti-rabbit secondary antibody (1:1000; Invitrogen).

The quantification of immunofluorescence (IF) was assessed using the Image J software. To quantify CPE IF levels, the fluorescence intensity of CPE staining was detected in each individual cell within the specified brain region. In the case of Iba1 or Aβ IF levels, we enumerated all intact cells within the designated pixel area in the indicated region of the brain.

Golgi-Cox staining

The hippocampal tissues of mice were immersed in the Golgi-Cox staining solution A/B from the FD Rapid Golgi Stain™ Kit (FD NeuroTechnologies, Shanghai, China) and incubated at room temperature for 14 days with careful protection from light. On the subsequent day, solution C was substituted, and the brain tissues were placed in this solution at 4 °C in darkness for 72 h. Subsequently, brain tissues were obtained as sections measuring 100 μm in thickness. These sections were then mounted on glass slides coated with gelatin and allowed to naturally dry for a period of 1 day. The Golgi-Cox reaction was carried out in accordance with the instructions provided by the manufacturer. Images were captured using a Leica fluorescence microscope (Leica TCS SP8 STED 3X, Germany) with objectives set at 200× and 640×. For each mouse, a total of 4-6 images were selected for analysis, and within each image, 50 to 70 neurons were examined, amounting to a comprehensive dataset across the study. The spine density was calculated by counting the number of spines along every 10 μm segment of the dendrites. This procedure was repeated for each neuron within the selected images.

Western blotting

The hippocampal tissues were lysed using RIPA buffer infused with protease inhibitors. To prepare the samples for electrophoresis, the lysates were heated to 95 °C for 10 min in the presence of Laemmli sample buffer containing sodium dodecyl sulfate (SDS) to ensure denaturation of the proteins. The samples were loaded onto 12% SDS-polyacrylamide gel electrophoresis (SDS-PAGE) gels and transfer to nitrocellulose membranes (0.45 μm; Millipore, Billerica, MA, USA). Subsequently, after being blocked with 5% non-fat milk, the membranes were probed with specific antibodies. These included anti-CPE antibody (1:1000; BD Bioscience), rabbit anti-PSD95 (1:1000; Cell Signaling Technology, Boston, USA), rabbit anti-Synapsin-1 (1:1000; Cell Signaling Technology), mouse anti-Aβ (1:500; Covance), mouse anti-GAPDH (1:1000, Cell

Signaling Technology), mouse anti-hAPP (1:1000, Biolegend, California, USA), and mouse anti-h+mAPP (1:1000, Biolegend). Following washing, the membranes were exposed to either anti-mouse or anti-rabbit secondary antibodies for a duration of one hour at room temperature. Bands were visualized and quantified using Image J software, with protein expression for each sample normalized to GAPDH.

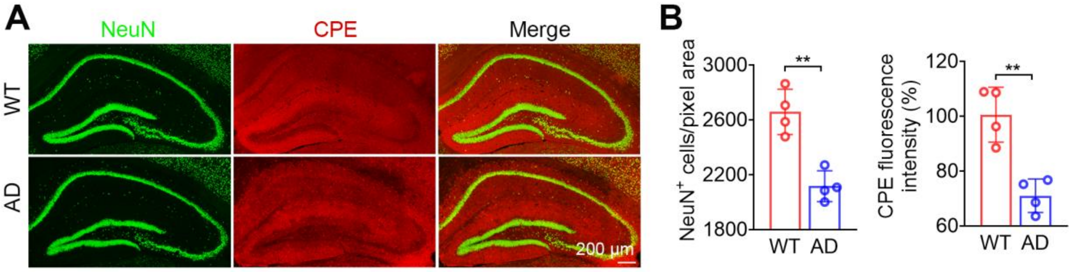

Figure S1. The levels of CPE are reduced in AD mice.

(A) Co-expression (NeuN and CPE) and quantification of NeuN and CPE in the hippocampus of 5-month-old wild type and 5×FAD mice.

(B) Quantification of $NeuN^+$ positive cells and CPE relative fluorescence in the hippocampus of 5-month-old wild type and 5×FAD mice, n = 4 mice, average of 4-6 slices per mouse, Unpaired t test, [Neuron: p = 0.0016; CPE: p = 0.0023].

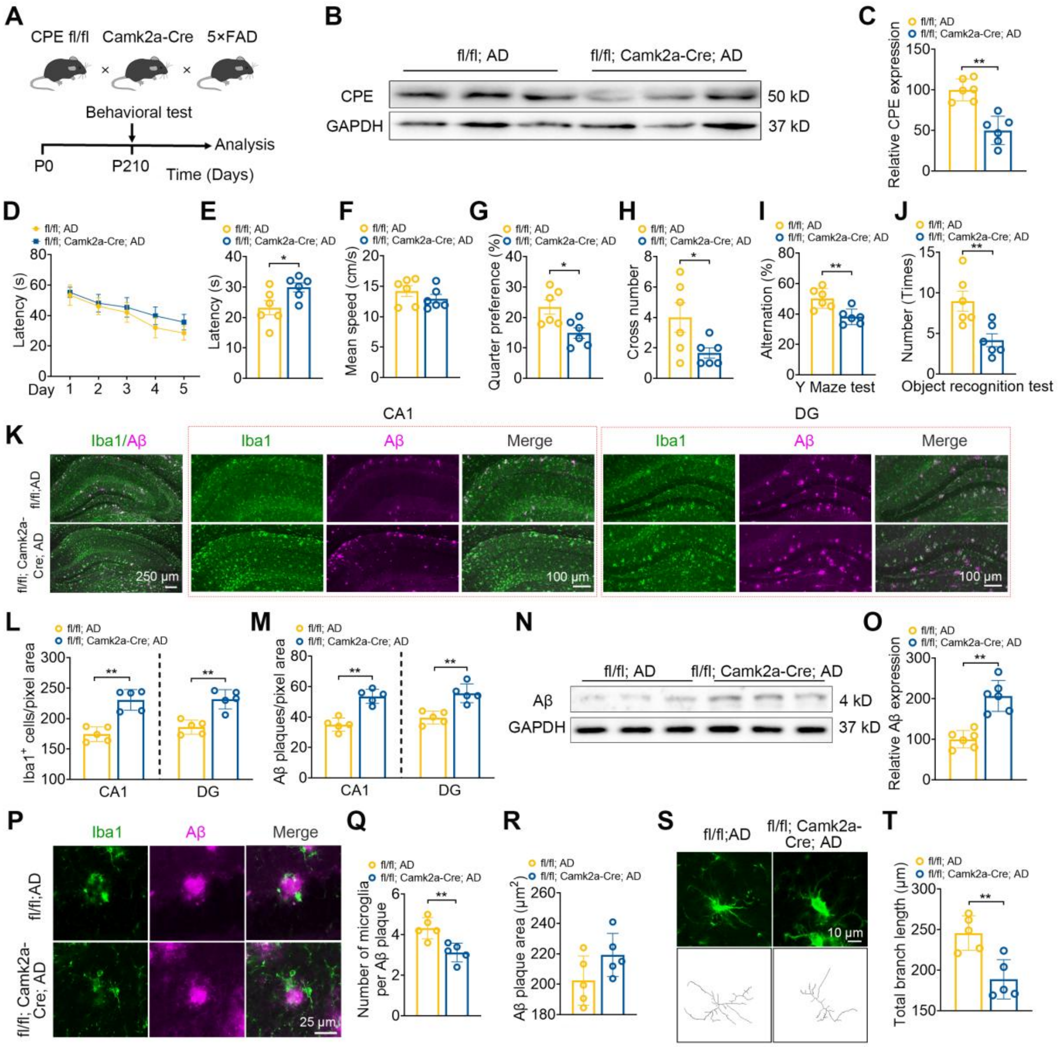

Figure S2. Neuronal CPE knockdown impairs cognitive functions, increases Aβ deposition, and elevates microglial numbers in 5×FAD mice at 7 months old.

(A) Schematic illustrating the generation of fl/fl: Camk2a-Cre: AD mice, along with behavioral testing. P0, postnatal day 0.

(B) Western blot analysis reveals the levels of CPE in the hippocampus of 7-month-old mice.

(C) CPE levels were normalized to GAPDH. n = 6 mice, Unpaired t test, [p = 0.0002].

(D) Morris Water Maze (MWM) analysis measures the latency (s) to reach the target in the invisible platform training.

(E) MWM analysis of latency (s), n = 6 mice, Unpaired t test, [p = 0.037].

(F) MWM Mean speed (centimeters per second), n = 6 mice, Unpaired t test, [p = 0.2906].

(G) MWM Target quarter preference (%), n = 6 mice, Unpaired t test, [$p = 0.015$].
(H) MWM Target cross number in the invisible platform tests, n = 6 mice, Unpaired t test, [$p = 0.0455$].
(I) Y maze test analysis as correct rate (%), n = 6 mice, Unpaired t test, [$p = 0.0049$].
(J) Object recognition test analysis as number (times), n = 6 mice, Unpaired t test, [$p = 0.0082$].
(K) Immunofluorescence was used to quantify the number of Iba1+ positive cells and Aβ plaque in the hippocampus of 7-month-old mice.
(L and M) Quantification of Iba1+ positive cells and Aβ plaque in the CA1 and dentate gyru. n = 5 mice, average of 4-6 slices per mouse, Unpaired t test, [CA1, Iba1+: $p = 0.0003$; DG, Iba1+: $p = 0.0008$; CA1, Aβ: $t = 6.552$, $p = 0.0002$]; DG, Aβ: Unpaired t test, [$p = 0.0014$].
(N and O) Immunofluorescent staining of Iba1 and Aβ, along with the statistical analysis of microglia number per Aβ plaque in fl/fl: AD and fl/fl: Camk2a-Cre: AD mice, n = 5 mice, average of 4-6 slices per mouse, Unpaired t test, [$p = 0.0048$].
(P-R) Immunofluorescent staining of Iba1 and the statistical analysis of total branch length (micrometers) and Aβ plaque size in fl/fl: AD and fl/fl: Camk2a-Cre: AD mice. n = 5 mice, average of 4-6 slices per mouse, average of 16 cells per slice, microglia number: Unpaired t test, [$p = 0.0042$], Aβ plaque size: Unpaired t test, [$p = 0.1192$].
(S) Western blot analysis demonstrates the levels of Aβ in the hippocampus of 7-month-old fl/fl: AD and fl/fl: Camk2a-Cre:AD mice.
(T) Aβ levels were normalized to GAPDH. n = 6 mice, Unpaired t test, [$p < 0.0001$].

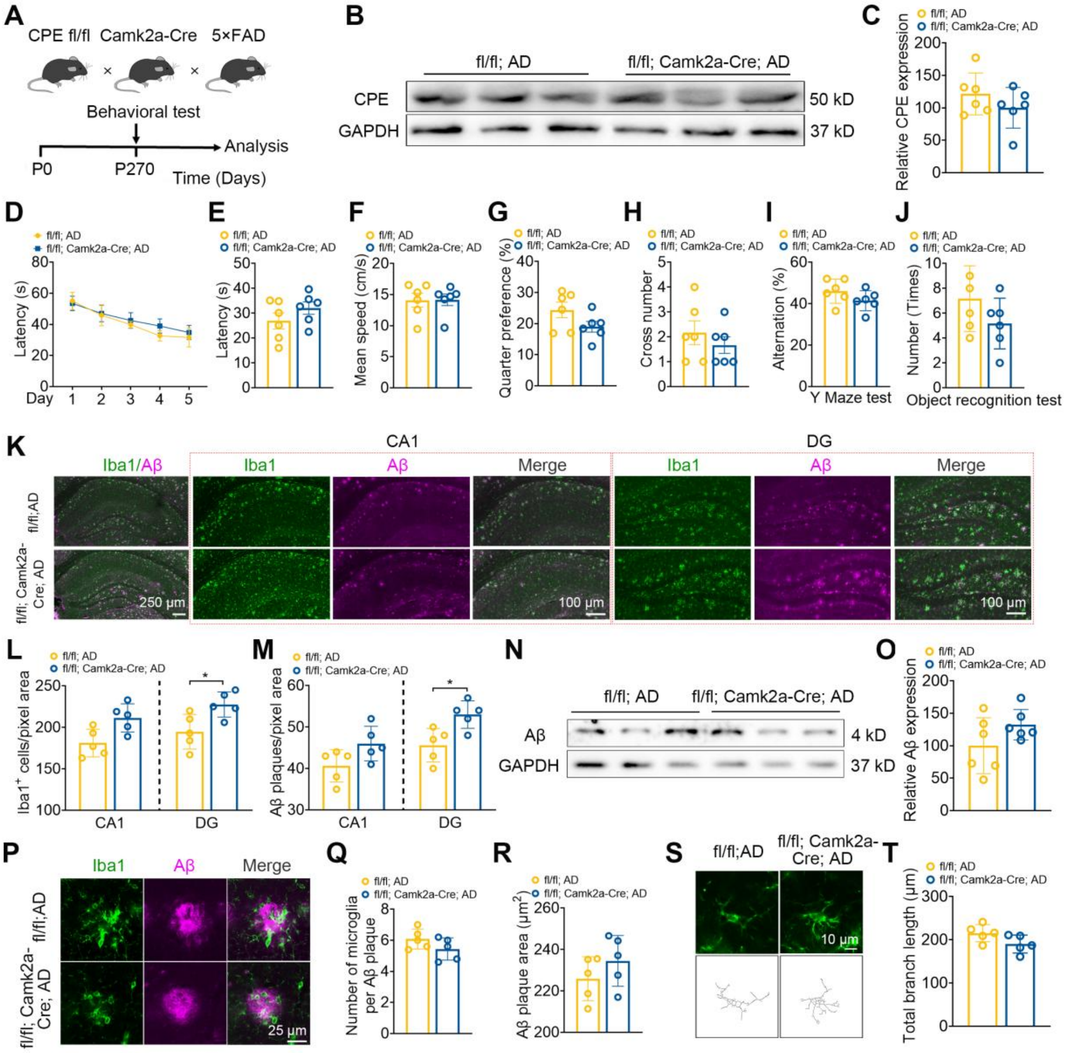

Figure S3. Neuronal CPE knockdown reduces synaptic proteins and modifies dendritic morphology in 5×FAD mice at 9 months old.

(A) Schematic illustrating the generation of fl/fl: Camk2a-Cre: AD mice, along with behavioral testing. P0, postnatal day 0.

(B) Western blot analysis reveals the levels of CPE in the hippocampus of 9-month-old mice.

(C) CPE levels were normalized to GAPDH. n = 6 mice, Unpaired t test, [p = 0.2696].

(D) Morris Water Maze (MWM) analysis measures the latency (s) to reach the target in the invisible platform training.

(E) MWM analysis of latency (s), n = 6 mice, Unpaired t test, [p = 0.2335].

(F) MWM Mean speed (centimeters per second), n = 6 mice, Unpaired t test, [p = 0.9605].

(G) MWM Target quarter preference (%), n = 6 mice, Unpaired t test, [p = 0.1037].

(H) MWM Target cross number in the invisible platform tests, n = 6 mice, Unpaired t test, [p = 0.4105].

(I) Y maze test analysis as correct rate (%), n = 6 mice, Unpaired t test, [p = 0.1673].

(J) Object recognition test analysis as number (times), n = 6 mice, Unpaired t test, [p = 0.1728].

(K) Immunofluorescence was used to quantify the number of Iba1+ positive cells and Aβ plaque in the hippocampus of 9-month-old mice.

(L and M) Quantification of Iba1+ positive cells and Aβ plaque in the CA1 and dentate gyrus. n = 5 mice, average of 4-6 slices per mouse, Unpaired t test, [CA1, Iba1+: p = 0.00798; DG, Iba1+: p = 0.0221]; Unpaired t test, [CA1, Aβ: p = 0.0680; DG, Aβ: p = 0.0133].

(N and O) Immunofluorescent staining of Iba1 and Aβ, along with the statistical analysis of microglia number per Aβ plaque in fl/fl: AD and fl/fl: Camk2a-Cre: AD mice, n = 5 mice, average of 4-6 slices per mouse, Unpaired t test, [p = 0.1740].

(P-R) Immunofluorescent staining of Iba1 and the statistical analysis of total branch length (micrometers) and Aβ plaque size in fl/fl: AD and fl/fl: Camk2a-Cre: AD mice. n = 5 mice, average of 4-6 slices per mouse, average of 16 cells per slice, microglia number: Unpaired t test, [p = 0.0831], Aβ plaque size: Unpaired t test, [p = 0.2681].

(S) Western blot analysis demonstrates the levels of Aβ in the hippocampus of 9-month-old fl/fl: AD and fl/fl: Camk2a-Cre:AD mice.

(T) Aβ levels were normalized to GAPDH. n = 6 mice, Unpaired t test, [p = 0.1368].

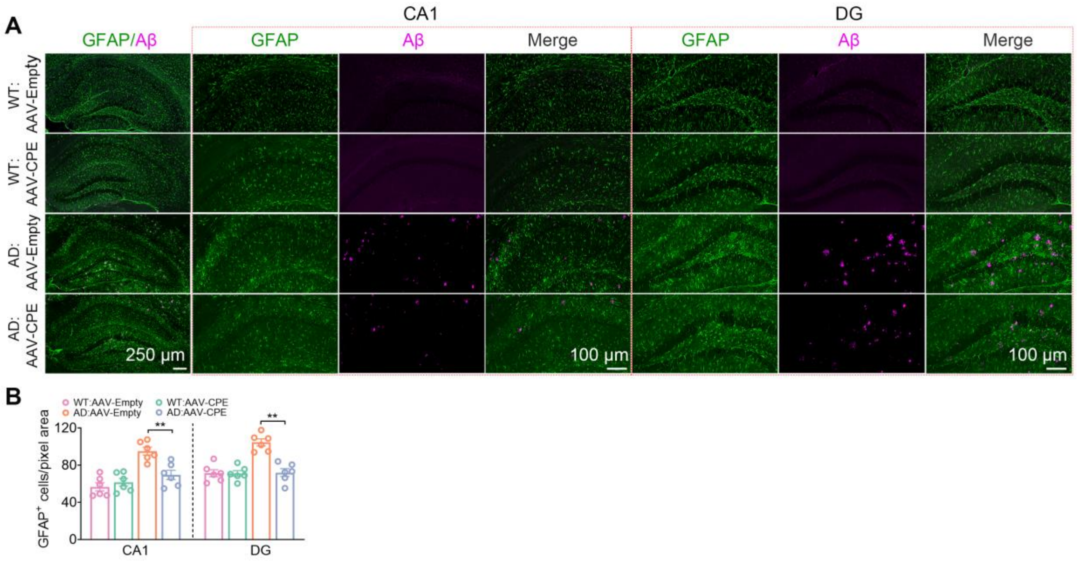

Figure S4. (A) Immunofluorescence was used to quantify the number of Iba1+ positive cells and Aβ plaques in the hippocampus of 5-month-old mice.

(B) Quantification of GFAP+ positive cells and Aβ plaques in the CA1 and dentate gyrus, n = 6 mice, averaged from 4-6 slices per mouse, One-way ANOVA analysis followed by Tukey's post-hoc multiple comparison test, CA1 [$F(3,20) = 14.93$, $p < 0.0001$; AD: AAV-Empty vs. AD: AAV-CPE, $p = 0.0029$]; DG, [$F(3, 20) = 19.36$, $p < 0.0001$; AD: AAV-Empty vs. AD: AAV-CPE, $p < 0.0001$]

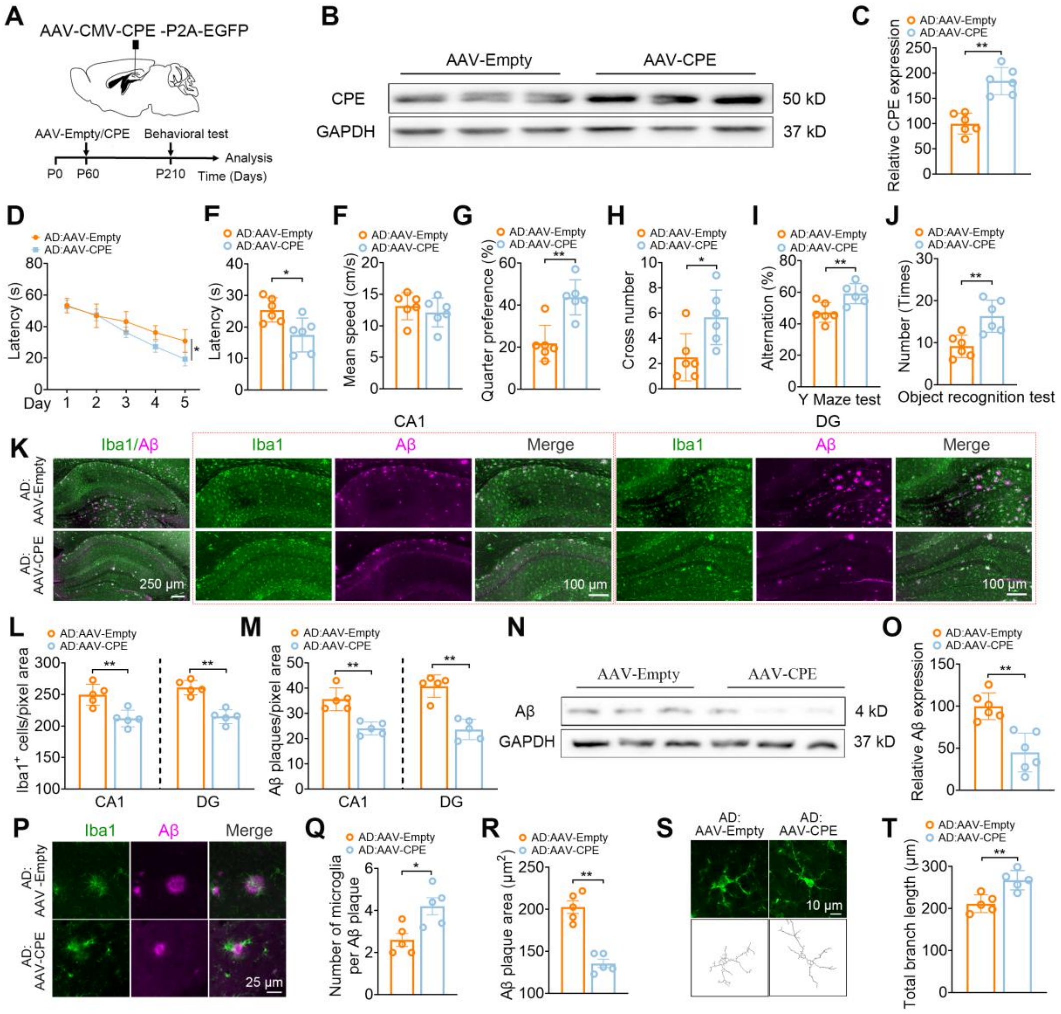

Figure S5. Enhanced CPE expression restores cognitive functions, reduces Aβ deposition, and diminishes microglial numbers in 5×FAD mice at 7 months old.

(A) Schematic depicting the administration of AAV-Empty/AAV-CPE, along with behavioral assessments. P0, the day of birth.

(B) Western blot analysis shows the levels of CPE in the hippocampus of 7-month-old mice.

(C) CPE levels were normalized to GAPDH. n = 6 mice, Unpaired t test, [$p < 0.0001$].

(D) Morris Water Maze (MWM) analysis determines the latency (s) to reach the invisible platform during training.

(E) MWM analysis assesses latency (s), n = 6 mice, Unpaired t test, [$p = 0.0146$].

(F) MWM: Mean speed (centimeters per second), n = 6 mice, Unpaired t test, [$p = 0.4582$].

(G) MWM: Target quarter preference (%), n = 6 mice, Unpaired t test, [p = 0.0012].

(H) MWM: Number of target crossings in the invisible platform tests, n = 6 mice, Unpaired t test, [p = 0.0218].

(I) Y maze test analysis as the percentage of correct choices, n = 6 mice, Unpaired t test, [p = 0.0086].

(J) Object recognition test analysis as the number of recognized objects, n = 6 mice, Unpaired t test, [p = 0.0036].

(K) Immunofluorescence was used to quantify the number of Iba1+ positive cells and Aβ plaques in the hippocampus of 7-month-old mice.

(L and M) Quantification of Iba1+ positive cells and Aβ plaques in the CA1 and dentate gyrus. n = 5 mice, averaged from 4-6 slices per mouse, Unpaired t test, [CA1, Iba1+: p = 0.0043; DG, Iba1+: t = 6.505, p = 0.0002]; Unpaired t test, [CA1, Aβ: p = 0.0010; DG, Aβ: p = 0.0002].

(N and O) Immunofluorescent staining of Iba1 and Aβ, along with the statistical analysis of the number of microglia per Aβ plaque in 5×FAD mice treated with AAV-Empty or AAV-CPE. n = 5 mice, average of 4-6 slices per mouse, Unpaired t test, [p = 0.0138].

(P-R) Immunofluorescent staining of Iba1 and the statistical analysis of total branch length (micrometers) and Aβ plaque size in 5×FAD mice treated with AAV-Empty or AAV-CPE. n = 5 mice, average of 4-6 slices per mouse, average of 16 cells per slice, microglia number: Unpaired t test, [p = 0.0040], Aβ plaque size: Unpaired t test, [p < 0.0001].

(S) Western blot analysis demonstrates the levels of Aβ in the hippocampus of 7-month-old 5×FAD mice treated with AAV-Empty or AAV-CPE.

(T) Aβ levels were normalized to GAPDH. n = 6 mice, Unpaired t test, [p = 0.0007].

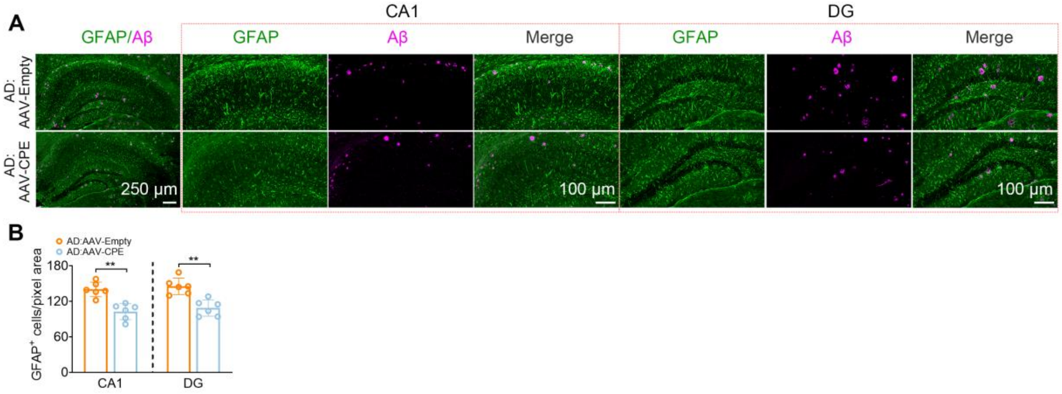

Figure S6. (A) Immunofluorescence was used to quantify the number of Iba1+ positive cells and Aβ plaques in the hippocampus of 7-month-old mice.

(B) Quantification of GFAP+ positive cells in the CA1 and dentate gyrus. n = 5 mice, averaged from 4-6 slices per mouse, Unpaired t test, [CA1: $p = 0.0022$; DG: $p = 0.0007$].

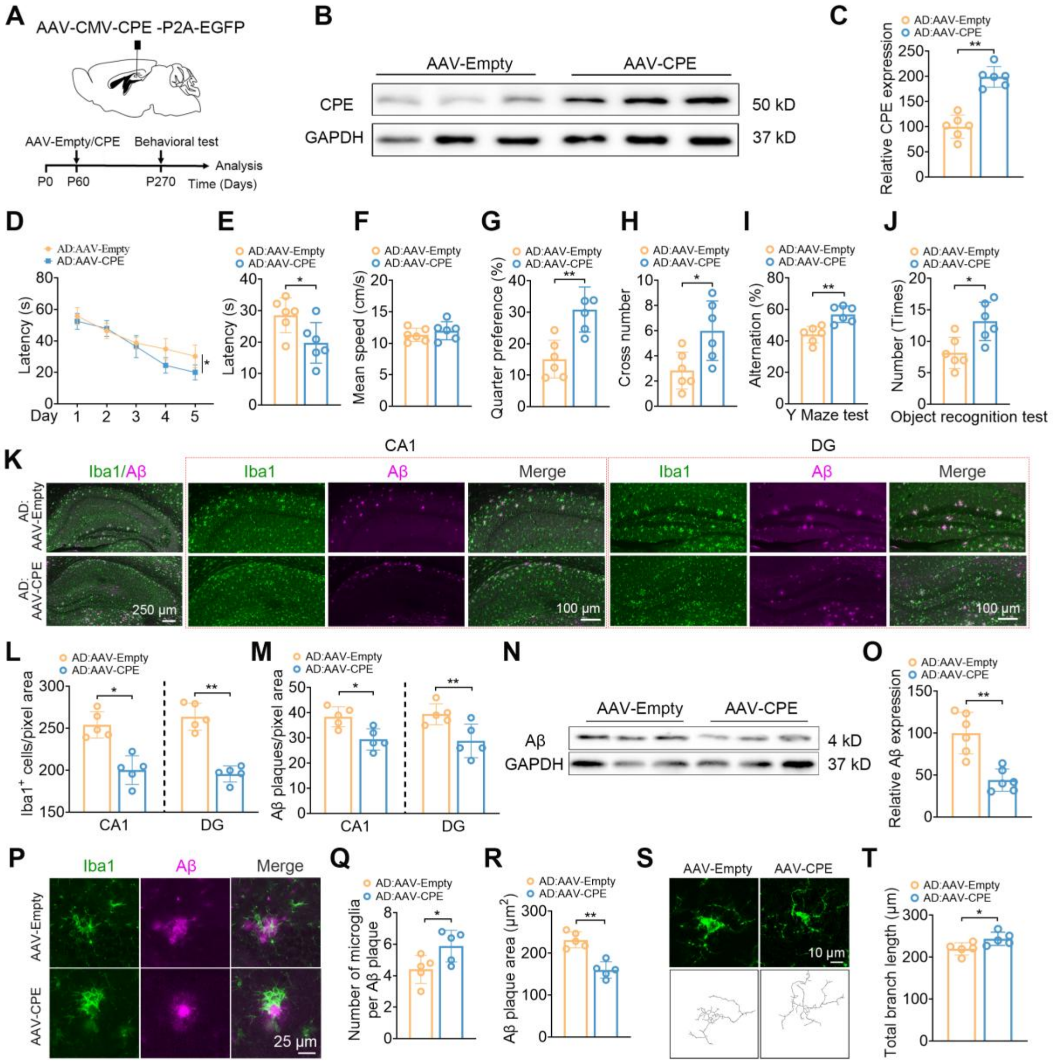

Figure S7. Enhanced CPE expression reinstates cognitive functions, reduces Aβ deposition, and diminishes microglial numbers in 5×FAD mice at 9 months old.

(A) Schematic depicting the administration of AAV-Empty/AAV-CPE, along with behavioral assessments. P0, the day of birth.

(B) Western blot analysis shows the levels of CPE in the hippocampus of 9-month-old mice.

(C) CPE levels were normalized to GAPDH. n = 6 mice, Unpaired t test, [$p < 0.0001$].

(D) Morris Water Maze (MWM) analysis determines the latency (s) to reach the invisible platform during training.

(E)MWM analysis assesses Latency (s), n = 6 mice, Unpaired t test, [$p = 0.0302$].

(F) MWM: Mean speed (centimeters per second), n = 6 mice, Unpaired t test, [p = 0.3884].

(G) MWM: Target quarter preference (%), n = 6 mice, Unpaired t test, [p = 0.0020].

(H) MWM: Number of target crossings in the invisible platform tests, n = 6 mice, Unpaired t test, [p = 0.0193].

(I) Y maze test analysis as the percentage of correct choices, n = 6 mice, Unpaired t test, [p = 0.0016].

(J) Object recognition test analysis as the number of recognized objects, n = 6 mice, Unpaired t test, [p = 0.0111].

(K) Immunofluorescence was used to quantify the number of Iba1+ positive cells and Aβ plaques in the hippocampus of 9-month-old mice.

(L and M) Quantification of Iba1+ positive cells and Aβ plaques in the CA1 and dentate gyrus. n = 5 mice, averaged from 4-6 slices per mouse, Unpaired t test, [CA1, Iba1+: p = 0.0090; DG, Iba1+: p = 0.0009]; Unpaired t test, [CA1, Aβ: p = 0.0168; DG, Aβ: p = 0.0084].

(N and O) Immunofluorescent staining of Iba1 and Aβ, along with the statistical analysis of the number of microglia per Aβ plaque in 5×FAD mice treated with AAV-Empty or AAV-CP. n = 5 mice, average of 4-6 slices per mouse, Unpaired t test, [p = 0.0394].

(P-R) Immunofluorescent staining of Iba1 and the statistical analysis of total branch length (micrometers) and Aβ plaque size in 5×FAD mice treated with AAV-Empty or AAV-CPE. n = 5 mice, average of 4-6 slices per mouse, average of 16 cells per slice, microglia number: Unpaired t test, [p = 0.0363], Aβ plaque size: Unpaired t test, [p = 0.0004].

(S) Western blot analysis demonstrates the levels of Aβ in the hippocampus of 7-month-old 5×FAD mice treated with AAV-Empty or AAV-CPE.

(T) Aβ levels were normalized to GAPDH. n = 6 mice, Unpaired t test, [p = 0.0006].

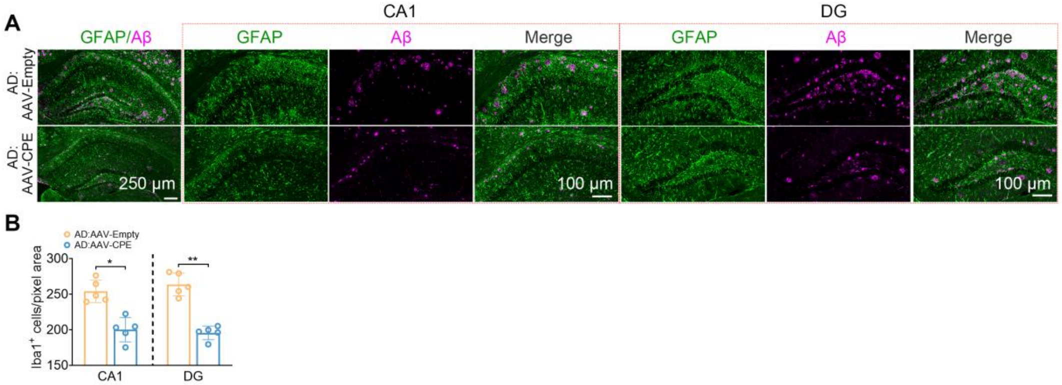

Figure S8. (A) Immunofluorescence was used to quantify the number of Iba1+ positive cells and Aβ plaques in the hippocampus of 9-month-old mice.

(B) Quantification of GFAP+ positive cells in the CA1 and dentate gyrus. n = 5 mice, averaged from 4-6 slices per mouse, Unpaired t test, [CA1: p = 0.0037; DG: p = 0.0002].
